# Probiotics and Fermented Foods in Human Nutrition

**DOI:** 10.3390/molecules31081353

**Published:** 2026-04-20

**Authors:** Irene Dini

**Affiliations:** Department of Pharmacy, University of Naples Federico II, 80131 Napoli, Italy; irdini@unina.it

**Keywords:** bioactive metabolites, nutraceutical development, microbial metabolism, postbiotics, food safety, food risk assessment

## Abstract

Fermented foods and probiotics represent complementary yet distinct components of human nutrition. Fermented foods are shaped by biochemical transformations driven by microbial metabolism, whereas probiotics are live microorganisms that may confer health benefits to the host. In both cases, bacteria, yeasts, and filamentous fungi mediate key metabolic activities that generate bioactive compounds and modulate host–microbiota interactions. During fermentation, microbial communities synthesize organic acids, peptides, exopolysaccharides, vitamins, and other metabolites that enhance food safety, sensory attributes, and potential health-promoting properties. Several microbial products, such as bacteriocins, reuterin, hydroxylated fatty acids, and exopolysaccharides, exhibit antimicrobial, immunomodulatory, antioxidant, and cholesterol-lowering activities. Advancing our understanding of microbial metabolism in fermented foods is essential for developing next-generation functional foods and nutraceuticals that leverage microbial biotransformations to support human health. Nonetheless, multiple challenges limit the translation of these advances into commercial products. Inadequately controlled fermentation may introduce microbiological or chemical hazards, regulatory frameworks governing microbial use in foods remain insufficiently defined, and standardized procedures for microbial strain handling and characterization are still lacking. This narrative review integrates current evidence on the nutraceutical properties of fermented foods and probiotics, while also examining the associated safety considerations and the technological factors that influence fermentation processes.

## 1. Introduction

**Figure 1:**
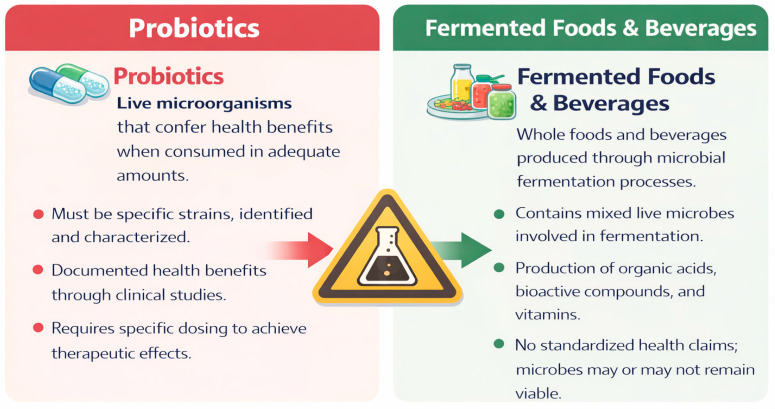
“Probiotics” vs. “fermented food or beverage”: key differences and overlaps. Image generated by Microsoft Copilot (April 2026).

Although commonly used interchangeably, probiotics, fermented foods, and fermented beverages represent distinct categories within contemporary food science and regulatory frameworks (Figure 1).

Probiotics, fermented foods, and fermented beverages are often conflated, yet they constitute distinct categories within food science and regulatory frameworks. Probiotics are live, clinically validated microorganisms by human studies that confer health benefits when consumed in adequate amounts, with effects that are strain-specific [1]. In contrast, fermented foods and beverages result from microbial transformations that enhance sensory, nutritional, and functional properties, but the viability and characterization of their microbial communities vary widely, and most strains have not undergone clinical evaluation [2].

This narrative review synthesizes current evidence on the nutraceutical properties of fermented foods and probiotics and examines safety considerations and technological factors influencing fermentation processes. To improve methodological transparency, the literature search for this narrative review followed a structured approach. Databases consulted included PubMed/MEDLINE, Scopus, Web of Science, and Google Scholar. Searches were conducted between January 2010 and February 2026 using predefined keyword combinations (“fermented foods”, “microbial metabolism”, “probiotics”, “postbiotics”, “lactic acid bacteria”, “food safety”, “microbial detoxification”). Inclusion criteria prioritized peer-reviewed studies addressing microbial metabolism, functional metabolites, safety aspects, and nutritional implications. Exclusion criteria included non-scientific sources, papers lacking methodological detail, and studies focused solely on industrial processing without biological relevance. Although not intended as a systematic review, this structured strategy reduces selection bias and enhances reproducibility.

## 2. Fermented Food and Beverage

Fermentation is one of the earliest biotechnological processes developed by human societies. It is obtained by the metabolic activities of bacteria, yeasts, and filamentous fungi that convert raw substrates into products with improved sensory, nutritional, and functional qualities [3,4]. Beyond enhancing preservation through mechanisms such as acidification, alcohol production, and microbial competition [5], fermentation can generate a wide range of bioactive metabolites with potential health benefits [6]. In recent years, the focus on fermented foods and beverages has shifted beyond the culinary domain toward their capacity to generate postbiotic compounds and support the development of functional foods. According to the ISAPP consensus, postbiotics are preparations of inanimate microorganisms and/or their components that confer a health benefit to the host. This definition includes fermentation-derived metabolites, cell-free supernatants, exopolysaccharides, peptides, and structurally modified phytochemicals, provided that their biological activity is demonstrated independently of microbial viability [7].

### 2.1. Microbial Ecology of Fermentation

Microbial fermentation is an anaerobic metabolic process in which microorganisms convert carbohydrate-rich substrates, including simple sugars, cereal grains, and other plant-derived materials, into alcohols, organic acids, and gaseous by-products [8]. The composition of microbial communities involved in fermentation varies substantially depending on the substrate, environmental conditions, and traditional or regional processing practices [9] (Table 1). Fermentation pathways can be categorized into lactic acid, alcoholic, acetic acid, and mold-based enzymatic fermentations.

Lactic acid fermentation converts carbohydrates into lactic acid via homofermentative or heterofermentative pathways, reducing pH and improving microbial safety [10]. Alcoholic fermentation relies on the glycolytic conversion of sugars into ethanol and carbon dioxide, accompanied by the formation of esters, higher alcohols, and other volatile compounds that shape the sensory profile of fermented beverages [11].

Acetic acid fermentation, via membrane-bound dehydrogenases, involves the oxidation of ethanol to acetic acid, which contributes to acidity and preservative effects [12].

Alkaline fermentation is characterized by extensive proteolysis, during which proteins are degraded into amino acids and peptides with the release of ammonia. The resulting increase in pH to around 8–9 inhibits spoilage microorganisms and imparts the characteristic umami aroma to foods such as nattō, traditionally fermented African legumes, and eggs. This process is primarily driven by *Bacillus* species and coagulase-negative *Staphylococcus*, which secrete extracellular proteinases responsible for proteolysis [13].

Mold-based fermentations produce foods with pronounced umami notes and complex textures, owing to extracellular proteases, amylases, and lipases that release amino acids, peptides, sugars, and aromatic compounds [14].

**Table 1:** Microbial ecology and metabolite profiles of major fermented food substrates.

Substrate	Examples	Dominant Microbes	Key Metabolites	Reference
Cereals	Sourdough, boza, and kvass.	*Aspergillus*, *Paecilomyces*, *Cladosporium*, *Penicillium, Fusarium*, and *Trichothecium*.	Organic acids, CO_2_, ethanol	[15,16,17]
Dairy	Kefir, yogurt.	Lactic acid bacteria, yeasts.	Lactic acid, peptides, exopolysaccharides	[18,19,20]
Legumes	Miso, tempeh, natto.	*Aspergillus oryzae*, *Mucor* spp. *Fusarium* spp., *Rhizopus*, *Bacillus subtilis.*	Amino acids, glutamate, isoflavone aglycones, and peptides	[21]
Meat/Fish	Fish sauce and salami.	Lactic acid bacteria, *Staphylococcus*, and halophiles.	Amino acids, peptides, organic acids, volatile esters, saturated alcohols	[22,23]
Vegetables	Sauerkrau, kimchi, pickles.	Lactic acid bacteria.	Lactic acid, vitamins, phenolic derivatives (flavonoids, polyphenols), glucosinolate breakdown products, and their derivatives.	[24]
Beverages	Cider, wine, beer, kombucha.	Yeasts, acetic acid bacteria, and lactic acid bacteria.	Ethanol, acetic acid, esters	[25,26]

### 2.2. Fermentative Microorganisms Contributing to Food Transformation

The diversity of microbial ecosystems involved in fermentation underpins the broad spectrum of biochemical processes responsible for the functional and sensory transformations observed in fermented foods.

#### 2.2.1. Lactic Acid Bacteria

Lactic acid bacteria (LAB), including *Lactobacillus, Streptococcus, Leuconostoc*, and *Pediococcus*, predominate in dairy, vegetable, and cereal fermentations, where they produce lactic acid and contribute to texture, flavor, and microbial safety.

These Gram-positive microorganisms can be non-spore-forming, aerotolerant, or facultatively anaerobic. Homofermentative LAB produce primarily lactic acid. Heterofermentative LAB generate lactic acid along with ethanol, CO_2_, acetic acid, and other metabolites [27].

According to the EFSA BIOHAZ Panel, food-associated LAB, including some antibiotic-resistant strains, rarely pose clinical risks, although they may serve as reservoirs of transmissible resistance genes within the food chain [28]. Most LAB species are classified as Generally Recognized as Safe (GRAS) due to their long history of safe use [29], and several taxa have received Qualified Presumption of Safety (QPS) status from EFSA [30].

Current market analyses project that the LAB sector will reach USD 2.22 billion by 2034 [31].

LAB comprise diverse genera and species used for specific technological functions such as enhancing microbial safety, extending shelf life, and improving sensory attributes in fermented foods [32,33].

They can produce proteolytic enzymes and a wide range of antimicrobial metabolites, including lactic and acetic acids, hydrogen peroxide, reuterin, exopolysaccharides (EPS), hydroxylated fatty acids, and bacteriocins that inhibit spoilage organisms and pathogens [34].

Organic acid production lowers pH, inhibits fungal growth, and contributes to mycotoxin mitigation [35]. Diacetyl disrupts microbial metabolism through reactions with amino groups, whereas hydrogen peroxide generates reactive oxygen species that damage fungal membranes and nucleic acids.

Reuterin, a multifunctional antimicrobial system produced by *Limosilactobacillus reuteri* during glycerol metabolism, exerts broad-spectrum inhibitory activity through multiple mechanisms. Reuterin alkylates thiol groups in microbial proteins and enzymes, disrupting redox homeostasis, impairing essential metabolic pathways, and inducing oxidative and electrophilic stress. This thiol-reactive activity leads to the inactivation of key enzymes involved in DNA synthesis, amino acid metabolism, and energy production, ultimately compromising cell viability. Although its synergistic action with lactic acid has been confirmed against bacteria, whether this interaction enhances antifungal efficacy remains unclear [36].

Fermentation by lactic acid bacteria reduces acrylamide levels by enabling its binding to cell wall components, thereby decreasing its bioavailability and overall concentration in food products [37].

Some *Lactiplantibacillus plantarum* strains can hydroxylate unsaturated fatty acids to form hydroxy derivatives, such as 3-hydroxytetradecanoic and 3-hydroxydodecanoic acids, able to disrupt fungal membranes and exert antifungal activity under the acidic conditions typical of fermented foods [29].

The EPS produced by LAB can have functional activities [38,39], support beneficial gut colonization [40], enhance probiotic persistence in the gastrointestinal tract [41], and support gut protection and cholesterol reduction [42].

Moreover, LAB can produce bacteriocins that act as antibacterial, antifungal, and antimycotoxigenic agents [43]. Due to their physicochemical stability and minimal impact on sensory attributes, bacteriocins are regarded as promising biopreservatives. Their antimicrobial activity is mediated through well-defined mechanisms, including pore formation in target cell membranes, disruption of the proton motive force, inhibition of cell wall biosynthesis, and interference with essential enzymatic pathways. Despite these advantages, their application in food systems may be limited by proteolytic degradation, uneven distribution within complex matrices, and interactions with food components that reduce bioavailability. These limitations can be mitigated through micro- and nanocapsulation strategies, which enhance stability and controlled release, or through their combination with complementary preservation technologies, such as mild heat treatments, high-pressure processing, or organic acid supplementation, to achieve synergistic antimicrobial effects [35]. The only bacteriocin approved for food use is Nisin, produced by *Lactococcus lactis*. It acts by binding lipid II and inducing pore formation in bacterial membranes [44]. It can limit Gram-positive bacteria and, indirectly, fungal growth by suppressing the growth of supportive bacteria [45].

LAB contribute to organoleptic development primarily through glycolysis, proteolysis, and, to a lesser extent, lipolysis [34]. Proteolysis is particularly relevant for flavor formation, generating amino acid-derived compounds such as alcohols, acids, esters, sulfur compounds, and aldehydes [46]. Some *Pediococcus* species, however, are associated with food spoilage [47].

#### 2.2.2. Acetic Acid Bacteria

Acetic acid bacteria (AAB), including *Acetobacter* and *Gluconobacter*, are Gram-negative bacteria that oxidize ethanol to acetic acid in aerobic conditions [12].

AAB are most active at 40–50 °C [48]. They are considered safe in traditional food fermentations; however, they are not included in the EFSA Qualified Presumption of Safety (QPS) list, cannot be automatically approved for use in novel foods or starter cultures, and therefore require strain-specific toxicological and genomic assessment [49]. AABs have significant industrial relevance, particularly in the production of vinegar, fermented beverages, and other oxidative fermentations, including sour beer, cocoa and coffee fermentations, kefir, kombucha, and sourdough [4]. AAB are obligately aerobic microorganisms capable of incompletely oxidizing sugars and alcohols into organic acids. Their metabolic activities also yield valuable compounds, including bacteriocins, extracellular polysaccharides, bacterial cellulose, aromatic metabolites, and essential coenzymes [26]. AAB can interact with co-occurring microorganisms to produce metabolites and drive the distinctive sensory profiles of fermented foods. Because these interactions vary with strain traits, nutrient availability, ecological conditions, and fermentation time, understanding them is essential for optimizing AAB performance in food processes [50].

#### 2.2.3. Yeasts

Yeast metabolism is crucial for establishing the thermal and biochemical conditions required for efficient fermentation. Through alcoholic fermentation, yeasts generate ethanol, CO_2_, and a diverse array of volatile metabolites, including higher alcohols, aldehydes, ketones, glycerol, and organic acids. The associated heat production, together with ethanol oxidation by acetic acid bacteria, maintains temperatures above 40 °C, a threshold essential for embryo inactivation, flavor-precursor formation, and proper color development [48]. Yeasts also degrade pectin in the pulp, improving aeration and supporting AAB growth [51]. Key yeast species include *Saccharomyces cerevisiae*, *Candida milleri*, *Pichia kudriavzevii*, and *Hanseniaspora opuntiae* [52].

*Saccharomyces cerevisiae* is highly fermentative and widely used as a starter culture to enhance fermentation efficiency and sensory quality of beer, wine, bread, sake, cider, and kefir.

*Candida milleri* contributes to the fermentation of kombucha, sourdough, and kefir [4].

*Pichia kudriavzevii* contributes aromatic compounds and tolerates high ethanol levels, while *Hanseniaspora opuntiae* produces esters associated with fruity notes [53].

*Debaryomyces hansenii* has been reported to limit or prevent the formation of biogenic amines by reducing the activity of decarboxylase-positive microorganisms [54].

Several yeasts have been evaluated as biological control agents against pathogenic bacteria. *Candida* spp., *Geotrichum candidum*, and *Pichia* spp. have exhibited inhibitory activity, particularly against *Listeria monocytogenes*.

Unfortunately, most evidence for yeast-mediated antibacterial effects derives from studies conducted in commercial media rather than in dry-cured fermented food matrices, so it is not known how they affect the technological or sensory properties of foods [55].

#### 2.2.4. Molds

Filamentous fungi such as *Aspergillus*, *Sordariaceae*, *Paecilomyces*, *Penicillium*, *Cladosporium*, and *Rhizopus* are central to the fermentation of many traditional foods, especially across Asia.

Molds can hydrolyze complex carbohydrates, proteins, and lipids through their powerful enzyme systems, thereby enhancing food flavor, texture, and nutrient bioavailability [56].

*Rhizopus*-derived phytases degrade phytates, thereby enhancing the bioavailability of minerals (zinc, iron, and calcium) [57]. The reduction in oligosaccharides such as stachyose and raffinose diminishes the flatulence-inducing properties of soy [58]. β-Glucosidases convert glycosylated polyphenols into their aglycones, which exhibit superior bioavailability and biological activity [59].

Mold fermentations can also generate beneficial metabolites, such as GABA, associated with relaxation and reduced anxiety [60], as well as the biosynthesis and release of polyphenols with antioxidant properties [17,61]. Examples of filamentous fungi that play a key role in traditional mold-based fermentations are *Aspergillus oryzae*, *Rhizopus oligosporus*, *Neurospora crassa*, *Penicillium roqueforti*, *Penicillium camemberti*, and *Monascus purpureus.*

Aspergillus oryzae drives the fermentation of soybeans and cereal substrates in traditional products such as soy sauce, miso, and sake [62,63]. *Rhizopus oligosporus* catalyzes the transformation of soy proteins into peptides and free amino acids during tempeh production [64], while *Neurospora crassa* contributes to specific soy-based fermentations [65]. In dairy systems, *Penicillium roqueforti* and *Penicillium camemberti* are responsible for the characteristic textures, flavors, and aromatic profiles of blue and soft-ripened cheeses [66]. Additionally, *Monascus purpureus* is employed in the production of red yeast rice, generating pigments and bioactive metabolites of technological and nutritional relevance [67].

#### 2.2.5. Mixed Fungal–Bacterial Consortia

**Figure 2:**
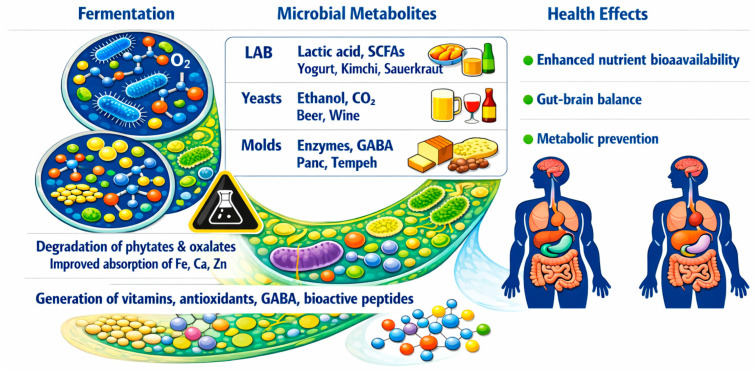
The fermentation frontier: how microbes transform foods in nutraceuticals. Image generated by Microsoft Copilot (April 2026).

Microbial consortia, structured communities of bacteria, yeasts, and filamentous fungi, exhibit synergistic interactions that markedly enhance plant-based fermentation. These mixed cultures efficiently depolymerize complex plant macromolecules, improve flavor and texture, and generate a wide array of bioactive metabolites, including polyphenols, peptides, carotenoids, and bacteriocins [68] (Figure 2). They also reduce antinutritional factors such as alkaloids, oxalates, and tannins [69].

Selected fungal strains can be employed as starter cultures to standardize production and accelerate fermentation. Traditional mixed fermentations, such as kefir and sourdough, rely on stable microbial consortia whose coordinated metabolic activities yield complex and distinctive metabolite profiles [70].

Co-culturing lactic acid bacteria with yeasts has been shown to extend shelf life and enhance sensory quality, while fungal partners contribute additional enzymatic functions that increase nutrient bioavailability [71].

Emerging strategies, including synthetic microbial communities, enable the rational design of simplified consortia that improve safety, limit the formation of undesirable compounds, and tailor the functional properties of fermented foods [71].

Advances in multi-omics technologies have further accelerated the characterization of these communities, supporting the selection of strains that enhance flavor, texture, and nutrient accessibility in plant-based matrices [72,73]. Moreover, genetic engineering and optimized bioreactor systems are driving the development of more efficient, scalable, and sustainable fermentation processes [74].

## 3. Nutritional and Functional Implications

Microorganisms can enhance nutrient bioavailability, detoxify foods contaminated with microbial toxins, mycotoxins, pesticides, heavy metals, and naturally occurring antinutritional factors. Moreover, they can generate a broad spectrum of bioactive compounds and modulate the host gut microbiota [6,7,8,9,10,11,12,13,14,15,16,17,18,19,20,21,22,23,24,25,26,27,28,29,30,31,32,33,34,35,36,37,38,39,40,41,42]. Health effects attributed to probiotics and microorganisms present in fermented foods are highly strain-specific and cannot be generalized at the species or genus level. Even closely related strains may differ substantially in their genomic profiles, metabolic outputs, and functional properties, including the production of organic acids, exopolysaccharides, bioactive peptides, and detoxifying enzymes. In addition, microbial behavior is strongly influenced by the food matrix: the same strain may exhibit different growth dynamics, enzymatic activities, and metabolite profiles in dairy, cereal, legume, or vegetable substrates. These matrix-dependent interactions further limit the extrapolation of findings across different fermented foods. To avoid overgeneralization, the revised manuscript explicitly distinguishes mechanistic observations from clinically validated outcomes and highlights the need for strain-resolved characterization when interpreting functional effects.

### 3.1. Improvements in Mineral and Protein Bioaccessibility Through Microbial Fermentation

Fermentation enhances protein digestibility by hydrolyzing proteins into peptides and free amino acids. This process is particularly relevant for legumes such as soybeans, which, despite their high protein content, contain antinutritional inhibitors that impair protein absorption. Fermentation with *Lactobacillus* species has been shown to enhance the digestibility and bioavailability of soybean proteins [75]. Comparable improvements have been reported in in vitro studies on fermented lentils, where fermentation significantly increased the proportion of digested protein [76]. Moreover, fermentation can improve mineral bioavailability. The limited bioavailability of minerals in plant-derived foods is largely attributed to their association with non-digestible compounds that form stable complexes resistant to human digestion. Fermentation can mitigate this limitation by degrading such complexes and releasing bound minerals, thereby improving their accessibility [77]. In vitro studies have reported increased levels of zinc, calcium, iron, and magnesium in fermented products, primarily due to the reduction in phytate content during microbial activity [78]. However, the apparent increase in mineral concentrations may also reflect dry-matter losses resulting from microbial degradation of proteins and carbohydrates during fermentation [79]. Additionally, in vitro evidence indicates that oxalate breakdown enhances the bioavailability of minerals, including iron, phosphorus, and calcium, by preventing the formation of insoluble complexes [80].

### 3.2. Mitigation of Microbial and Chemical Food Contaminants Through Microbial Fermentation

LAB can detoxify foodborne hazards through a combination of microbiological and chemical mechanisms that target both biological toxins and chemical contaminants.

Microbiological detoxification is largely mediated by the antimicrobial activity of metabolites produced by lactic acid bacteria, including lactic acid, short-chain fatty acids, hydrogen peroxide, carbonyl compounds, and bacteriocins. These metabolites inhibit or inactivate toxigenic microorganisms such as Escherichia coli, Listeria monocytogenes, Salmonella, and Clostridium botulinum by disrupting membrane integrity, collapsing proton gradients, interfering with metabolic pathways, and suppressing the expression of virulence genes. Surface-associated proteins and competitive adhesion mechanisms further limit pathogen colonization [81].

Detoxification of major mycotoxins by LAB occurs through two principal mechanisms cell-wall adsorption and enzymatic or metabolic biotransformation.

Adsorption is a rapid and reversible process in which mycotoxins bind non-covalently to structural components of the microbial cell wall. In LAB, these include thick peptidoglycan layers enriched in polysaccharides, teichoic acids, and S-layer proteins, whose negatively charged functional groups enhance binding affinity. In yeasts such as Saccharomyces, β(1,3)D-glucans and mannoproteins play a central role in toxin sequestration, with adsorption efficiency determined by cell wall architecture and glucan organization. This mechanism is strain-dependent and influenced by toxin structure and environmental conditions [82].

Biodegradation involves the enzymatic conversion of mycotoxins into less toxic metabolites. Although less extensively characterized, in vitro studies indicate that several probiotic strains, including *Pediococcus pentosaceus*, *Bacillus licheniformis*, and *Saccharomyces pastorianus*, are capable of degrading aflatoxin B1, zearalenone, deoxynivalenol, T-2 toxin, HT-2 toxin, and other trichothecenes through enzymatic pathways, often mediated by extracellular enzymes that are sensitive to heat and proteolysis [83,84,85,86].

Lactic acid bacteria can also detoxify pesticides through two principal mechanisms: enzymatic biodegradation and cell-surface-mediated biosorption.

Hydrolytic enzymes such as carboxylesterases, organophosphate hydrolases, phosphotriesterases, and phosphatases to cleave and metabolize organochlorine, organophosphate, neonicotinoid, and pyrethroid pesticides into less toxic derivatives [87]. Several strains, including *Leuconostoc mesenteroides*, *Lactiplantibacillus plantarum*, *Lactobacillus sakei*, and *Levilactobacillus brevis*, can utilize pesticides as carbon and energy sources, with degradation efficiencies often associated with specific genes such as *opdB*, which encodes an esterase containing the conserved ‘Gly-X-Ser-X-Gly’ motif characteristic of organophosphate-degrading enzymes [88].

LAB can mitigate heavy metal toxicity primarily through: biosorption (rapid, metabolism-independent) and bioaccumulation (slower, metabolism-dependent).

Biosorption is a rapid, metabolism-independent process in which metal cations bind to negatively charged functional groups, carboxyl, phosphate, and hydroxyl, located on peptidoglycan, teichoic acids, S-layer proteins, and exopolysaccharides of the LAB cell wall [89]. Bioaccumulation is slower and requires metabolic activity, allowing metals to cross the cell membrane and accumulate intracellularly once surface binding sites become saturated. Both mechanisms are strongly strain-specific and are influenced by environmental pH [90].

### 3.3. Mitigation of Antinutritional Compounds Through Microbial Fermentation

Microbial fermentation offers an effective strategy to mitigate the impact of antinutritional factors, including phytic acid, tannins, lectins, saponins, and protease inhibitors, plant-derived compounds that hinder nutrient digestion and absorption [91] (Table 2).

**Table 2:** Main classes of antinutrients and their mechanisms of action.

Antinutrient	Mechanism of Action	Physiological/Nutritional Effects	References
Tannins	Water-soluble phenolics form insoluble complexes with proteins and metal ions	Reduced protein digestibility and mineral bioavailability	[92]
Oxalates	Present as soluble oxalic acid and insoluble calcium oxalate; chelate minerals	Reduced mineral absorption; soluble oxalates may crystallize in the kidneys (nephrolithiasis)	[93]
Phytates (phytic acid)	Chelate divalent and trivalent cations (Fe^2+^, Fe^3+^, Cu^2+^, Mg^2+^, Ca^2+^, Zn^2+^); bind proteins and starches	Marked reduction in mineral, protein, and starch bioavailability	[94]
Protease inhibitors (PPIs)	Inhibit digestive enzymes (trypsin, chymotrypsin)	Impaired protein digestion; reduced availability of sulfur amino acids (Met, Cys); pancreatic hypertrophy/hyperplasia	[95,96]
Lectins	Carbohydrate-binding glycoproteins resistant to digestion; bind epithelial cells	Gastrointestinal irritation; immune activation; associated with IBS, Crohn’s disease, leaky gut; insulin mimicry; NLRP3 activation	[97]
Cyanogenic glycosides	Hydrolyzed by β-glucosidases → release hydrogen cyanide (HCN), inhibitor of cellular respiration	Acute/chronic cyanide toxicity; metabolic acidosis; growth impairment	[98,99,100,101]
Goitrogenic glycosides (glucosinolates)	Interfere with iodine uptake and thyroid hormone synthesis	Potential risk of hypothyroidism; evidence is inconsistent in balanced diets	[102,103]
Saponins	Amphiphilic glycosides form complexes with membrane sterols (cholesterol)	Hemolysis increase membrane permeability, hemoglobin leakage); effects vary by aglycone and sugar composition	[104,105]

**Figure 3:**
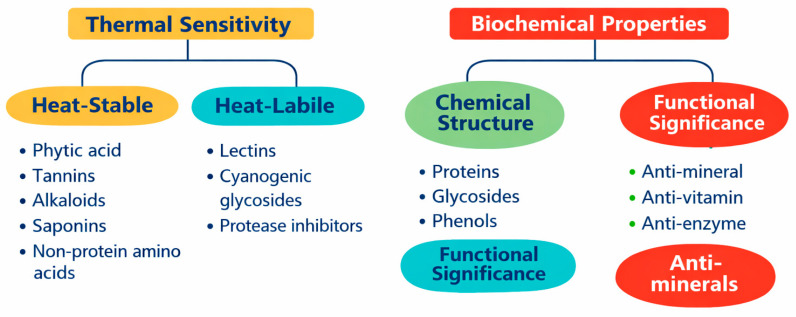
Classification of antinutritional factors. Image generated by Microsoft Copilot (April 2026).

Antinutritional factors can be classified according to their thermal sensitivity (heat-stable vs. heat-labile), chemical structure (proteins, glycosides, phenolics), and functional effects (anti-mineral, anti-vitamin, anti-enzyme) (Figure 3). From a thermal standpoint, they include heat-stable compounds, such as phytic acid, tannins, alkaloids, saponins, and non-protein amino acids, as well as heat-labile compounds, including lectins, cyanogenic glycosides, and protease inhibitors. A complementary classification considers their chemical structure (e.g., proteins, glycosides, phenolic compounds) and their functional effects, distinguishing factors with anti-mineral, anti-vitamin, or anti-enzyme activities [106].

During fermentation, enzymes such as phytases, tannases, oxalate decarboxylases, and β-glucosidases may be released, enabling the degradation of antinutritional factors. Phytases hydrolyze phytic acid into inorganic phosphate and lower myo-inositol esters, thereby improving the availability of minerals such as Ca^2+^ and Mg^2+^ [107]. Tannases cleave ester and depside bonds in tannins, generating gallic acid, ellagic acid, and glucose. The gallate decarboxylase generated by *Lactiplantibacillus plantarum* can convert gallic acid into pyrogallol [108]. Oxalate decarboxylases produced by *Lactobacillus plantarum* and *Lactobacillus acidophilus* can catalyze the decarboxylation of oxalate to CO_2_, reducing oxalate content in fermented matrices [108]. β-Glucosidases hydrolyze saponin glycosides, decreasing saponin solubility and toxicity, and can also promote cyanide release from cyanogenic glycosides through cleavage of β-glycosidic bonds [97].

### 3.4. Bioactive Metabolites Derived from Fermentation and Their Physiological Roles

Fermented foods can act not only as nutrient sources but also as functional carriers of microbiota-driven bioactivity, reinforcing their relevance in personalized nutrition and functional food development.

Lactic acid bacteria, yeasts, and other probiotic strains can interact with the host gut microbiota, modulating its composition and metabolic activity [109,110]. Through the bioconversion of complex carbohydrates, proteins, lipids, and other dietary substrates, the resident and introduced microbes generate a wide spectrum of bioactive compounds, including short-chain fatty acids (SCFAs), bioactive peptides, EPS, and vitamins [109,111,112,113].

SCFAs, in particular, play a central role in gut–immune crosstalk modulating immune cell function and promoting anti-inflammatory cytokine production [114]. Butyrate also supports intestinal barrier integrity by serving as a primary energy source for colonocytes and reducing gut permeability [115]. Beyond local effects, SCFAs and other microbial metabolites influence systemic metabolic pathways, oxidative stress responses, and gut–brain axis signaling, contributing to improved cognitive function, stress resilience, and mental well-being [116,117,118].

EPS can exhibit multiple functional effects, including antioxidant, immunomodulatory, antibiofilm, and cholesterol-lowering activities [38,39]. They also promote beneficial gut colonization and probiotic persistence by enhancing biofilm formation, limiting pathogen adhesion, and protecting epithelial cells [40,41]. Additionally, exopolysaccharides (EPS) can contribute to gut health by binding dietary toxins and reducing their bioavailability, and they may also lower cholesterol levels by enhancing bile acid secretion [42].

## 4. Safety and Quality Considerations

Fermentation entails intrinsic risks when not properly managed. These risks primarily involve microbiological and chemical hazards that may compromise product safety. Health effects attributed to probiotics are strain-specific and cannot be generalized at the species level. Functional properties of fermented foods often derive from matrix-dependent interactions rather than microbial viability alone. Many bioactive metabolites (e.g., EPS, bacteriocins, peptides) have demonstrated activity primarily in vitro or in animal models, and clinically validated effects remain limited. This clarification prevents overgeneralization of mechanistic findings.

### 4.1. Microbiological Hazards

A major safety concern in fermented foods is contamination by pathogenic microorganisms. Although fermentation relies on beneficial microbes, such as LAB, to inhibit pathogens, including *Listeria monocytogenes*, *Escherichia coli*, *Salmonella*, and *Clostridium botulinum*, they may persist if fermentation parameters are not adequately controlled [119]. Documented cases include *Listeria* contamination in soft cheeses [120] and *Clostridium botulinum* outbreaks associated with improperly fermented fish or vegetable products [121,122]. Traditional mitigation requires strict hygiene practices, maintenance of appropriate temperature, acidity, anaerobic conditions, the use of starter cultures with established safety profiles, thermal pasteurization, and synthetic preservatives [123]. Conventional approaches, although effective in ensuring safety, often disrupt beneficial microbiota and compromise sensory and regional characteristics of fermented products [124,125,126,127,128,129]. Emerging technologies, including cold plasma, plasma-activated water, in-package plasma systems, electromagnetic wave–based treatments (photodynamic inactivation, pulsed light, catalytic infrared radiation, microwave, and radio-frequency heating), and natural essential oils, offer targeted antimicrobial effects [24,25,26,27,28,29,30,31,32,33,34,35,36,37,38,39,40].

Cold plasma and its derivatives, such as plasma-activated water, plasma-activated interfaces, and in-package plasma, selectively inhibit Gram-negative and nitrite-producing bacteria through RONS (molecules generated during cold plasma treatments, including reactive oxygen species (ROS) such as O_2_^−^, OH∙, H_2_O_2_, and reactive nitrogen species (RNS) such as NO∙, NO_2_^−^, and NO_3_^−^), membrane disruption, and differences in antioxidant defenses, thereby improving nitrite control and enhancing sensory quality during fermentation [130,131,132].

Cold plasma exhibits varying levels of selective inactivation depending on the vegetable’s surface morphology. Smooth root and stem vegetables allow microorganisms to remain fully exposed, making plasma action more efficient, whereas leafy vegetables with deep folds create protective niches that limit plasma penetration. As a result, treatment parameters must be specifically adjusted for each type of vegetable to ensure effective microbial control while preserving desirable fermentative microbiota [123].

Photodynamic inactivation (PDI) and pulsed light (PLS) exploit ROS generation and photophysical effects to inactivate pathogens with minimal thermal damage. PDI technology provides non-thermal antimicrobial action, leaves no chemical residues, and requires low energy input [133]. PDI can inhibit or eliminate Gram-positive (*Listeria monocytogenes)*, Gram-negative (*Escherichia coli*, *Pseudomonas aeruginosa*), mold (*Aspergillus flavus*, *Fusarium avenaceum*, *Trichotecium roseum*, *Rhizopus oryzae*), yeast (*Staphilococcus cerevisiae*, *Rhodotorula mucilaginosa*, *Kloeckera javanica)*, and filamentous fungi (*Alternaria alternata, S. cerevisiae*) [134].

PLS combines photothermal and photochemical actions that selectively damage microorganisms, but its UV-C component, responsible for most of the inactivation, penetrates only shallowly into solid matrices, allowing microbes hidden in surface folds to survive. PLS exhibits a broad antimicrobial spectrum, effectively targeting Gram-negative bacteria (*Salmonella enterica*, *Escherichia coli*), Gram-positive bacteria (*Listeria monocytogenes*, *Bacillus cereus*), yeasts (*Saccharomyces cerevisiae*), and molds (*Aspergillus niger*, *Penicillium roqueforti*) [135,136].

Catalytic infrared radiation (CIR), microwave heating, and radio-frequency treatments all provide selective thermal or thermo-electromagnetic inactivation by exploiting differences among microbes in moisture content and electromagnetic sensitivity. CIR uses mid-infrared radiation (3.3–3.8 µm) generated by Pt–Pd catalytic combustion to induce protein denaturation, DNA melting, membrane phase transitions, and peptidoglycan cleavage, achieving efficient and clean microbial reduction while sparing low-moisture vegetable tissues; this selectivity is enhanced by the strong water-gradient effect, which concentrates infrared energy in high-moisture microbial cells [137,138,139].

Microwave sterilization combines rapid volumetric heating with non-thermal electromagnetic effects that alter membrane potential and ion permeability, producing differential susceptibility among microbial groups and enabling the inactivation of resistant genera such as *Bacillus*, *Clostridium*, *Penicillium*, and *Aspergillus* under optimized frequency and field-strength conditions [140,141].

Radio-frequency heating (3 kHz–300 MHz) enables deep, uniform heat penetration through dipole rotation and ion migration. This treatment disrupts membrane homeostasis, interferes with cell wall biosynthesis, and impairs energy metabolism, effectively targeting both Gram-positive and Gram-negative bacteria. Owing to its rapid heating rates, volumetric energy transfer, and compatibility with continuous-flow systems, radio-frequency heating is well suited for industrial-scale applications [142].

Moreover, several non-pathogenic members of fermentation microbiota (e.g., lactic acid bacteria, coagulase-negative *Staphylococci, Enterococci*) can carry intrinsic or acquired resistance traits, and horizontal gene transfer (HGT) may be facilitated by the high cell density, close intercellular proximity, biofilm formation, and abundance of mobile genetic elements characteristic of fermentation ecosystems [143,144]. Meta-analyses and culture- and metagenomic-based investigations have identified ARGs and viable resistant isolates in selected retail products (e.g., kimchi, artisan cheeses) and reported that diets enriched in fermented foods may transiently elevate gut resistome levels in healthy adults. However, the clinical implications of these shifts remain uncertain and appear to be context dependent [145]. Conversely, substrate-anchored metagenomic analyses across a wide range of fermentations (including kombucha and water kefir) reveal substantial variability among products, with several plant-based ferments exhibiting low or even undetectable ARG levels under the conditions evaluated [146]. The LAB’s ability to acquire and transfer mobile AR determinants was documented, with several studies reporting horizontal gene transfer (HGT) events both among *Lactobacillus* species and between *Lactobacillus* and pathogenic bacteria. Notably, in vitro and in vivo studies showed that vancomycin resistance can be transferred from Enterococci of human and animal origin to *Lactobacillus acidophilus* in mice [147]. Given the high frequency of such events in animal models, similar mechanisms are likely to occur in the human gastrointestinal tract, which is recognized as a major reservoir for ARGs [148].

Enterococci, frequently detected in animal-derived fermented products, combine intrinsic and acquired antimicrobial resistance with the ability to withstand low pH, high salt concentrations, and elevated temperatures, enabling their persistence throughout fermentation. Horizontal transfer of resistance determinants has been documented, including the transmission of the tetracycline resistance gene *tet(M)* from *Enterococcus faecium* isolated from dry sausage to clinical isolates of *Enterococcus faecalis* and *Enterococcus faecium*, mediated by a type-1 integron [143,149]. The potential dissemination of antimicrobial resistance genes (ARGs) by non-pathogenic members of the fermentation microbiota can be mitigated through a combination of strain selection, process control, and targeted monitoring. The use of well-characterized starter cultures with established safety profiles and lacking mobile genetic elements represents an important safeguard [150]. Advances in whole-genome sequencing enable the exclusion of strains carrying plasmids, transposons, or integrative conjugative elements associated with transmissible resistance, thereby reducing the likelihood of HGT [151].

Maintaining strict hygiene and environmental controls throughout production further prevents the introduction of resistant contaminants such as *Enterococcus* spp., which are known to persist under fermentation conditions and to transfer resistance determinants (e.g., *tet(M)*) to other bacteria [152].

Additionally, controlled fermentation parameters, including pH, salinity, temperature, and anaerobiosis, limit the proliferation of opportunistic microorganisms capable of participating in HGT and reduce the survival of pathogens such as *Listeria monocytogenes*, *Escherichia coli*, *Salmonella*, and *Clostridium botulinum* [4].

Finally, routine surveillance programs integrating metagenomics, qPCR-based ARG detection, and periodic verification of starter culture integrity can be used [143].

### 4.2. Chemical Hazards in Fermented Foods

Chemical hazards in fermented foods arise primarily from the accumulation of biogenic amines (BAs), such as histamine, tyramine, cadaverine, and putrescine, produced through microbial decarboxylation of amino acids [153]. Elevated BA levels can cause adverse reactions, including headaches, hypertension, nausea, and allergic responses, especially in individuals with reduced metabolic capacity for these compounds [119].

Histamine poisoning is frequently linked to fermented fish products, where inadequate fermentation or storage can result in toxic concentrations [154].

Tyramine, commonly present in fermented cheeses and certain alcoholic beverages, may trigger hypertensive crises in individuals taking monoamine oxidase inhibitors [155]. Regulatory agencies have established maximum allowable BA levels in high-risk fermented foods. Risk management strategies include controlling fermentation parameters (temperature, pH, and duration), selecting microbial strains with low decarboxylase activity, and ensuring proper post-fermentation storage [14].

## 5. Traditional vs. Industrial Fermentation

Both traditional and modern fermentation extend the shelf life of perishable raw materials [156]. Traditional fermentations are generally spontaneous processes driven by the indigenous microbiota naturally present in raw materials and the surrounding environment. These conditions give rise to products characterized by high microbial diversity and distinctive regional traits. Several authors suggest that fermentation practices and their derived products originated approximately 10,000 years ago, primarily as strategies to extend food shelf life during periods of scarcity while also enhancing sensory qualities [13,157]. In ancient societies, fermentation occurred without deliberate inoculation, relying instead on naturally occurring microorganisms associated with the substrate and its environment [158]. These complex microbial consortia, comprising bacteria, yeasts, and filamentous fungi, transform substrates and contribute to the characteristic flavors, aromas, and textures of traditional fermented products [159].

By contrast, modern approaches employ selected starter cultures, engineered microbial consortia, and genetically modified or synthetically designed strains to achieve predictable acidification, flavor formation, antioxidant production, enzyme production, pigment production, vitamin production, and inhibition of spoilage organisms [160,161,162,163]. Advances in genetic engineering and synthetic biology now enable microorganisms to efficiently synthesize targeted metabolites, including amino acids, organic acids, enzymes, and functional proteins, such as recombinant caseins used for animal-free cheese production [160,164].

Precision fermentation boosts the development of next-generation animal-free foods. Several companies produce milk, egg, and meat analogs through engineered yeasts and filamentous fungi that replicate the techno-functional properties of animal proteins [165]. Artificial intelligence has further accelerated ingredient design and fermentation optimization, exemplified by platforms that match plant matrices to the molecular profiles of animal products [166].

Recent research has used precision fermentation to produce sauces, powders, and pastes with sensory properties comparable to those of conventional seafood-based products, starting from edible insects [167,168].

The global precision fermentation market is projected to grow from USD 2.8 billion in 2023 to over USD 36 billion by 2030 [169].

### 5.1. Modern Fermentation and Its Contribution to Food Sustainability

Modern fermentation improves sustainability by supporting circularity, reducing waste by converting food waste into novel bioactive ingredients [170], enhancing resource efficiency, and enabling the development of novel, nutrient-dense food products [171]. Precision fermentation and microbial biomass production require substantially less energy and water than conventional livestock or crop-based protein systems, as microorganisms efficiently convert substrates into high-value nutrients with minimal spatial demands [172]. These processes are also associated with lower greenhouse gas emissions [173], offering a viable alternative to ruminant agriculture and other emission-intensive production models. Advances in bioreactor engineering, metabolic optimization, and process control have further improved the energy efficiency of fermentation-based bioprocesses [174]. Fermentation plays a key role in the valorization of agricultural and food waste by transforming low-value side streams, such as whey, fruit pomace, and cereal bran, into functional ingredients, organic acids, bioactive peptides, and microbial biomass, thereby supporting circular bioeconomy strategies and reducing the volume of organic waste requiring disposal [175]. Controlled fermentation strengthens food security and resilience by supporting decentralized, scalable production systems that are less dependent on climate-sensitive agriculture, while rapid microbial growth cycles help stabilize supply chains [69]. Diversifying protein sources through fermentation reduces vulnerability to environmental and geopolitical disruptions [176]. Additional sustainability benefits include reduced antibiotic use, as fermentation-derived enzymes and probiotics can replace antimicrobials in animal feed [177]. Finally, fermentation serves as a versatile platform technology for producing bio-based materials such as bioplastics and biopolymers, indirectly contributing to sustainable packaging solutions and reducing dependence on petrochemical resources [178].

**Figure 4:**
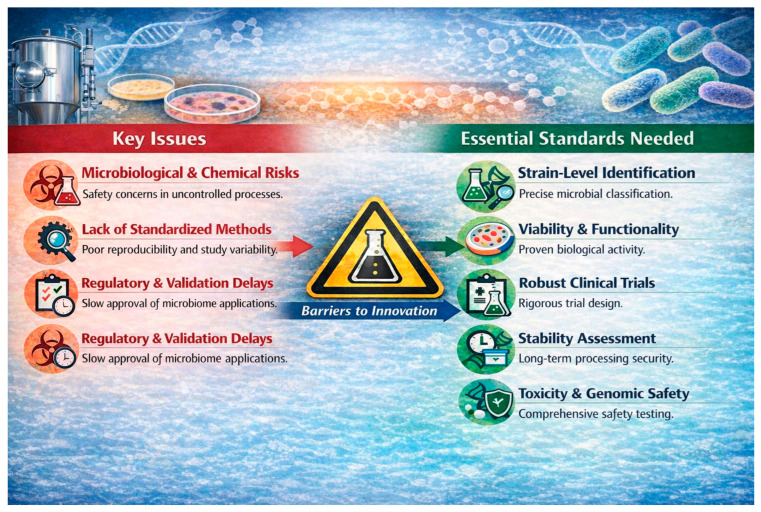
Modern fermentation challenge for effective application. The figure was generated using NotebookLM (Gemini 1.5 model), which was consulted in February 2026.

Persistent research and regulatory gaps continue to limit the integration of microbiome-derived innovations into food systems (Figure 4). A major barrier is the lack of standardized methods for isolating, characterizing, and applying microbial strains and consortia, which undermines reproducibility, complicates cross-study comparisons, and slows both clinical validation and regulatory approval [166,179]. Harmonized international guidelines are needed to define essential parameters, including accurate taxonomic and strain-level identification, assessment of cell viability and in vivo bioactivity, rigorous randomized clinical trial design, evaluation of long-term stability during processing and storage, and comprehensive toxicological and genomic safety testing [180].

### 5.2. Consumer Perceptions

Consumer acceptance of fermentation technologies is shaped by a wide range of interconnected factors, with perceptions of naturalness emerging as a central determinant. Traditional fermentation is generally viewed as familiar, historically rooted, and therefore more natural and safer, whereas precision fermentation, despite its sustainability potential, may be perceived as less natural or unfamiliar [181,182]. These perceptions contribute to broader acceptance challenges, including concerns about technological complexity, genetic modification, food safety, and allergenicity [183,184]. Technology neophobia further affects both traditional and precision fermentation: while some consumers express apprehension toward live microbes and contamination risks in traditional ferments [185,186], precision fermentation elicits uncertainty due to its novelty, biotechnological basis, and unfamiliar production processes [161,187]. Concerns about genetic modification remain particularly salient, as precision fermentation often relies on engineered microorganisms, and skepticism toward GMOs, rooted in perceived health and environmental risks, can extend to these products [188]. Although precision-fermented ingredients may be compositionally identical to traditional ones, consumers may still question their safety, long-term effects, and ecological implications [189].

Ethical and societal considerations also play a role, including worries about the displacement of traditional farming, job losses, and the broader implications of high-tech food systems. Cultural and emotional attachments to traditional food practices, along with concerns about transparency, trust, and regulatory oversight, further shape consumer responses. Economic factors add another layer of complexity: perceptions of naturalness strongly influence purchasing behavior and market viability [190], while fears of industry monopolization, pricing, and accessibility contribute to skepticism [191,192].

Moreover, the current high production costs of precision-fermented ingredients pose challenges for affordability and widespread adoption.

Addressing these multifaceted concerns requires transparent communication, inclusive public engagement, robust regulatory frameworks, and evidence-based information to support informed consumer understanding and foster confidence in precision fermentation.

## 6. Fermented Foods and Beverage

Although documentation is limited, more than 5000 fermented products are known worldwide [193]. Cultural and geographical factors influenced the substrates used for traditional fermented foods. In the Middle East, Europe, and India, fermentations use mainly milk as a substrate, whereas in Asian regions, where animal husbandry is historically more limited, rice, grains, vegetables, fish, and soybeans are used. In Africa, millet, sorghum, maize, and wheat are the predominant substrates for fermentation [194].

### 6.1. Fermented Foods and Beverages from Africa

Africa has a long tradition of fermenting plant and animal-based foods, typically produced at the household level under rudimentary, often unhygienic conditions. This knowledge is largely transmitted orally and preserved by elderly rural women. Sour milk is the most widely described fermented food in Africa. These foods fall into three broad categories: foodstuffs (cereals, tubers, vegetables), relishes/condiments/sauces, and beverages (alcoholic and non-alcoholic) (Table 3). The lack of a written tradition complicates historical reconstruction, yet fermented foods are central to African cultural identity and gastronomy [193].

#### 6.1.1. Soubala

Soumbala is produced through the alkaline fermentation of *Parkia biglobosa* seeds and is widely consumed across West Africa for its nutritional and functional properties [195]. Fermentation enhances the bioavailability of essential amino acids and fatty acids, making soumbala a nutrient-dense food [196]. This process also reduces antinutritional factors and their associated allergenic effects, improving digestibility and overall safety [196,197]. Due to its low sugar content, soumbala is recommended for individuals with diabetes [197]. Its richness in unsaturated fatty acids and phenolic compounds contributes to the prevention of cardiovascular diseases and other oxidative-stress-related disorders [198]. Additionally, it provides dietary fiber and minerals that may help combat nutritional deficiencies [199]. Regular consumption has been associated with reduced risks of hypertension, diabetes, and obesity [200,201]. Although most studies focus on raw soumbala, it is commonly consumed after cooking, which can degrade heat-sensitive nutrients, phenolics, flavonoids, and microbial enzymes, thereby diminishing its health benefits [199,202]. To preserve its nutritional value, raw soumbala should be encouraged, yet its strong sensory characteristics limit its acceptance [196]. A promising strategy is the development of a soumbala-based seasoning enriched with locally used spices, such as onion, pepper, parsley, and green anise, which can improve flavor while providing additional anti-inflammatory, antimicrobial, antioxidant, and anticancer properties [183,203,204,205].

#### 6.1.2. Bikalga

Bikalga is a traditional, alkaline-fermented condiment made from *Hibiscus sabdariffa* seeds, widely consumed in West and Central Africa. Its processing involves prolonged cooking with alkalinizing ash leachate followed by spontaneous fermentation dominated by *Bacillus* species, particularly *B. subtilis* [206]. Fermentation induces extensive proteolysis, lipolysis, and carbohydrate degradation, thereby improving digestibility and enhancing nutrient bioavailability. Notably, Bikalga retains high protein and lipid levels [207]. The process also leads to a substantial increase in mineral content, especially potassium, sodium, calcium, and sulfur, due to both microbial metabolism and the mineral-rich ash leachate [207]. Beyond its nutritional benefits, Bikalga is traditionally valued for its medicinal uses, including the management of high blood pressure, diarrhea, and infections, suggesting potential antimicrobial effects due to *Bacillus* spp. [207].

#### 6.1.3. Dawadawa

Dawadawa is a condiment produced by alkaline fermentation of *Parkia biglobosa* seeds. It is a culturally and nutritionally important food across West Africa. Fermentation profoundly enhances its nutraceutical value through proteolysis, lipid modification, the release of bioactive phenolic compounds, and increased antioxidant activity. Nutritionally, dawadawa provides 35–43% protein, a complete essential amino acid profile that meets FAO/WHO requirements, and substantial levels of calcium, iron, zinc, magnesium, and phosphorus. These features underscore its relevance as a functional food that supports metabolic health, immune function, and nutrient adequacy in populations with limited access to animal protein. Functional properties such as favorable water and oil absorption capacities further support its incorporation into diverse food formulations. Local processing practices influence its nutraceutical quality [208].

#### 6.1.4. Okpehe

Okpehe is a traditional fermented condiment made from *Prosopis africana* seeds consumed across several West African regions. Its nutraceutical relevance stems from the biochemical transformations that occur during alkaline fermentation by autochthonous *Bacillus* species. Proteolysis hydrolyzes seed proteins into peptides, amino acids, and ammonia, thereby enhancing digestibility and contributing to the product’s characteristic aroma [209]. Amylolytic activity increases the availability of soluble sugars, and selected strains of *Bacillus subtilis* synthesize polyglutamic acid (PGA), a compound associated with improved texture, viscosity, and bioactive functions [210]. Okpehe also contains bioactive peptides and volatile compounds that may exert antioxidant, antimicrobial, and gut-modulating effects [210]. The use of carefully selected starter cultures, particularly *Bacillus subtilis* strains BFE 5301 and BFE 5372, enhances both safety and quality. Notably, strain BFE 5301 produces subtilisin, a heat-stable bacteriocin active at acidic pH values and capable of inhibiting *Bacillus cereus*, thereby improving microbiological safety during fermentation [211].

#### 6.1.5. Ugba

The African “Ugba” or “Ukpaka” oil obtained from *Pentaclethra macrophylla (P. macrophylla) Benth* bean seed, is traditionally used in the preparation of the popular African salad. The seeds require extensive cooking followed by fermentation to ensure palatability and enhance nutrient bioavailability. Fermented *Pentaclethra macrophylla* seeds have been associated with several health-promoting properties, as demonstrated by in vivo studies in animal models, including anti-anemic effects [212], anti-hyperlipidemic activity [213], and blood-pressure-lowering potential [214]. They also serve as a valuable source of phytochemicals and essential nutritional elements [215]. Phytochemical investigations have identified bioactive secondary metabolites such as the diterpene secopentaclethrolide [16α,17-dihydroxy-6,7-seco-ent-kaurane-19,6β-olide] and the 1,3-diglyceride 1-arachidonyl-3-linoleoyl-sn-glycerol, further underscoring the nutraceutical potential of this traditionally fermented legume. Finally, fermented *P. macrophylla* seeds offer significant protection against diabetes-associated neurological disorders through combined antioxidant, anti-inflammatory, and enzyme-modulating mechanisms. Specifically, its use significantly reduces the activities of acetylcholinesterase, butyrylcholinesterase, angiotensin-converting enzyme, and arginase, key biochemical markers implicated in neurodegeneration and metabolic dysfunction. Finally, as evidenced by in vivo studies in diabetic rats, ugba markedly enhances brain antioxidant levels, elevates thiol concentrations, increases superoxide dismutase and catalase activities, and reduces malondialdehyde equivalents [216].

#### 6.1.6. Iru

Iru is a traditional fermented condiment produced from the seeds of the African locust bean (*Parkia biglobosa*). Widely consumed across West Africa and particularly appreciated among the Yoruba people of Nigeria, it is commonly incorporated into traditional dishes such as melon soup, okra soup, and various vegetable-based preparations [217], *Bacillus* spp. (*B. encimensis*, *B. safensis*), *Enterococcus* (*E. dispar*) and *Lysinibacillus* spp. (*L. fusiformis*, *L. macroides*), represent the key bacteria responsible for the fermentation of *iru* [218]. Over the course of fermentation, crude fat, ash, and fiber contents increase [219]. Processing methods markedly shape both the nutritional profile and sensory attributes of *iru*. Chemical catalysts negatively affect the nutritive quality of iru. Comparative analyses of traditional and no-potash iru show that *iru* produced without chemical additives and no potash methods retains the highest levels of essential nutrients. Sensory characteristics are strongly method-dependent: steaming preserves the creamy color of the cotyledons, minimizes off-odors by preventing direct water contact with the testa, yields a slightly marshy but clean-tasting product, and permits superior hygiene. In contrast, water-based cooking promotes higher dehulling efficiency but increases absorption of undesirable flavors and alters texture through greater hydration of the cotyledons. Overall, steaming offers the most favorable balance of nutritional retention, sensory quality, and processing hygiene, making it a recommended method for high-quality, scalable *iru* production [220]. Recent findings suggest that fermenting *P. biglobosa* seeds with selected bacterial strains may constrain dyslipidemia by reducing total cholesterol, LDL cholesterol, and triglycerides. Notably, several formulations demonstrated lipid-lowering effects that surpassed those of conventional statin therapy, as evidenced by in vivo studies conducted in Wistar rats [221].

#### 6.1.7. Kawal

Kawal is a traditional fermented food condiment produced through natural alkaline fermentation due to the spore-forming *Bacillus* of the leaves of *Senna obtusifolia* (also known as *Cassia obtusifolia*). The process begins with harvesting mature leaves at the end of the rainy season, then cleaning and pounding them into a paste, which is placed in earthenware jars and buried for 15 days to allow fermentation. Kawal provides antimicrobial compounds (surfactins, iturins, fengycins, and kurstakins) with potential applications in food bioprotection and agriculture [222].

#### 6.1.8. Sigda

Sigda is a traditional Sudanese fermented food produced from sesame (*Sesamum indicum* L.) oilseed cake, a process that markedly enhances its palatability and nutritional value. The preparation involves pounding the seedcake with warm water, allowing the mixture to ferment for 3–7 days at about 30 °C in sealed earthenware or metal vessels, sometimes supplemented with kambo (an alkaline ash extract), and shaping the mixture into small balls for long-term storage. The fermentation is driven primarily by a homofermentative lactic acid bacterium (*Streptococcus* sp.), which dominates the microflora and produces substantial amounts of lactic acid, up to “50 g kg^−1^ during fermentation”, resulting in a rapid decline in pH to approximately 4.0 within three days. This acidification contributes to microbial safety and improved digestibility. Nutritionally, Sigda retains the high protein content of sesame seedcake (approximately 440–460 g kg^−1^) and preserves its rich sulfur amino acid profile. Fermentation increases the levels of leucine, isoleucine, and alanine [223].

#### 6.1.9. Furundu

Furundu is a traditional Sudanese fermented food made from crushed seeds of Hibiscus sabdariffa (karkade) through spontaneous fermentation, primarily driven by *Bacillus* species. This fermentation transforms the raw seeds, which are naturally rich in sulfur-containing amino acids but otherwise unpalatable, into a nutritionally valuable, shelf-stable food. Fermentation preserves and enhances the biological quality of the seed proteins, improves digestibility, reduces antinutritional factors, and generates a meat-like flavor highly appreciated by local populations [223].

#### 6.1.10. Umqombothi

Umqombothi is a nutrient-dense traditional opaque sorghum beer produced through spontaneous fermentation of a mixture of sorghum malt, maize meal, and maize malt, with promising functional properties. Its preparation involves steeping and malting sorghum, mixing it with maize meal, cooking the mixture, and allowing it to undergo natural fermentation driven by lactic acid bacteria and yeasts, a process that shapes its characteristic flavor, low alcohol content (2–3.5% *v*/*v*), and nutrient profile. Umqombothi contains dietary fiber, essential minerals (Na, Mg, K, P, Fe, Zn, Ca, S), amino acids such as glutamic acid and leucine, B-group vitamins (including nicotinic acid and folate), and soluble sugars such as maltose and glucose. Controlled fermentation has been shown to improve its nutritional value, increasing crude protein, total amino acids, nicotinic acid, ash content, and bioactive metabolites, while reducing antinutritional factors such as phytates. Moreover, sorghum fermentation produces phenolic acids, flavonoids, phytosterols, and antioxidant compounds [224].

#### 6.1.11. Burukutu

Burukutu is a traditional Nigerian fermented beverage made from sorghum or millet enriched with gari, a fermented cassava product. Its production involves steeping and germinating sorghum grains, drying and milling the malt, boiling the flour–water mixture, adding gari, and then spontaneous fermentation for 2–3 days. Fermentation increases crude protein content while reducing sugars and ascorbic acid, and it enhances amino acid availability, particularly lysine, methionine, and serine, although heat-sensitive amino acids such as cystine and tryptophan are lost. The final beverage contains simple sugars (glucose, fructose, sucrose, maltose) and reaches an alcohol content of about 4%. Overall, burukutu provides easily digestible nutrients that support energy and growth. Fermentation improves flavor, digestibility, and protein quality [225].

#### 6.1.12. Dégué

Dégué is a traditional West African fermented cereal product typically prepared from pearl millet, which is cleaned, soaked, germinated, dried, milled, and then mixed with milk or dairy components before undergoing spontaneous or controlled fermentation. Fermentation of millet reduces antinutritional factors such as phytic acid (−78%) and polyphenols (−46%), while enhancing protein content, protein digestibility, starch digestibility, and mineral bioavailability, particularly calcium, iron, magnesium, and zinc. The process also increases free phosphorus and decreases phytate-bound phosphorus, further improving mineral absorption [226].

#### 6.1.13. Ogi

Ogi is a traditional West African fermented cereal porridge made from maize, sorghum, or millet through a sequence of steeping, wet milling, sieving, and spontaneous lactic fermentation. The grains are typically soaked for 24–72 h to allow natural microbial activity, then wet-milled and sieved to remove bran and fiber, after which the slurry is left to ferment for 1–3 days, yielding a mildly acidic, smooth paste. The use of *Lactobacillus* spp. and *Saccharomyces* spp. enhances fermentation efficiency, improves safety, and increases nutritional quality by accelerating acidification, reducing pathogenic load, and promoting the degradation of antinutritional factors such as phytates and polyphenols. Controlled fermentation also increases the bioavailability of essential minerals (Ca, Fe, Zn), improves protein digestibility, and enriches the product with B-group vitamins and beneficial metabolites. As a result, ogi prepared with defined starter cultures offers superior nutritional value, enhanced microbial safety, and more consistent sensory quality compared with spontaneously fermented ogi [227].

#### 6.1.14. Gari

Gari is a fermented, free-flowing granular product derived from cassava and widely consumed across Nigeria, where it serves as a major carbohydrate source and is commonly reconstituted with hot water to form a stiff dough known as *Eba*. The process involves peeling and washing cassava roots, grating them into a mash, allowing spontaneous lactic fermentation to occur, then dewatering, sieving, and toasting to obtain the final granular product. Fermentation plays a crucial role in improving the safety and quality of gari by reducing the potential toxicity of cassava cyanogenic compounds and modifying the physicochemical characteristics of the starch matrix. Fermentation, together with the incorporation of fermented maize residues, alters water absorption, swelling power, solubility, and pasting behavior, largely due to insoluble dietary fiber and partially degraded starch structures. These biochemical modifications also influence nutritional attributes, increasing protein, fat, fiber, and mineral content while reducing carbohydrate concentration and glycemic index [228].

#### 6.1.15. Togwa

Togwa is a traditional fermented beverage consumed in Tanzanian households, commonly used as a weaning food or as a refreshing drink. It is produced from the short fermentation of the finger millet and maize flour. Its preparation begins with cleaning and steeping maize kernels in water for 1–3 days to allow natural fermentation, followed by wet milling and sieving to separate the starchy slurry from the fibrous residue. The slurry is then allowed to sediment, after which the supernatant is decanted, and the fermented starch is collected [229]. This spontaneous lactic acid fermentation improves the product’s microbial safety, enhances nutrient bioavailability, and contributes desirable sensory attributes. Because Togwa relies on spontaneous fermentation, product quality can vary considerably. Fermentation reduces antinutritional factors such as phytates and polyphenols, increases mineral accessibility, and improves protein digestibility, while the dominance of lactic acid bacteria (e.g., *Lactobacillus plantarum*, *Lactobacillus fermentum*, *Pediococcus pentosaceus*) contributes probiotic potential and extended shelf-life [230].

#### 6.1.16. Bushera

Bushera is the most produced traditional non-alcoholic beverage in Uganda. *Lactococcus*, *Lactobacillus*, *Leuconostoc*, *Enterococcus*, and *Weissella* are LAB employed in *bushera* production. It is typically prepared at the household level by low-income women, using sorghum and millet, both for home consumption and for small-scale sale. Its fermentation is frequently accelerated by back-slopping, resulting in a more pronounced acidity. In local practice, Bushera at different fermentation stages serves distinct consumer groups: the mildly fermented Day-1 product is commonly given to young children, whereas adults prefer the fully fermented beverage. Bushera, as a natural probiotic source, may support digestion, enhance nutrient uptake, and promote gut health [230].

#### 6.1.17. Shamita

Shamita is a traditional Ethiopian cereal-based fermented beverage produced primarily from roasted barley flour mixed with water to form a slurry, which is then subjected to spontaneous lactic acid fermentation. The process typically involves mixing the flour with lukewarm water, adding small quantities of spices, and allowing the mixture to ferment for 12–24 h at ambient temperature, during which naturally occurring LAB dominate the microbial community. This fermentation reduces pH, enhances microbial safety, and contributes to the beverage’s characteristic sour flavor. Nutritionally, Shamita provides carbohydrates, dietary fiber, and moderate amounts of protein, while fermentation increases the bioavailability of micronutrients by reducing antinutritional factors such as phytates. Moreover, the proliferation of LAB, particularly species of *Lactobacillus*, *Pediococcus*, and *Leuconostoc*, confers potential probiotic benefits, including improved gut microbial balance, production of short-chain fatty acids, and modulation of immune responses [231].

#### 6.1.18. Munkoyo

Munkoyo is a traditional Zambian non-alcoholic fermented beverage produced from maize porridge and Rhynchosia root extracts, used as both an enzymatic source and a microbial inoculum. Production begins by mixing maize flour with water and cooking the mixture for approximately 30 min to gelatinize the starch; after cooling, *Rhynchosia* roots are added to hydrolyze complex carbohydrates and initiate spontaneous fermentation, which proceeds for about 48 h at ambient temperature in vessels such as calabashes or metal drums. The fermentation is driven predominantly by lactic acid bacteria (*Lactobacillus*, *Streptococcus, Lactococcus*, *Klebsiella, Enterobacter*, and *Acetobacter*), typically four to ten species, which acidify the matrix and contribute to product safety and stability. Nutritionally, Munkoyo provides carbohydrates, small amounts of protein, and B-vitamins. Fermentation enhances micronutrient bioavailability by reducing antinutritional factors such as phytates. The dominance of LAB also confers potential probiotic benefits, including modulation of the gut microbiota, production of organic acids, and possible contributions to immune and metabolic health [232].

#### 6.1.19. Mahewu

Mahewu is a traditional Southern African non-alcoholic fermented beverage produced from maize porridge that undergoes spontaneous or inoculated lactic acid fermentation. Its preparation typically involves cooking maize meal into a thin porridge, cooling it to lukewarm, and introducing either natural back-slopping material or specific inocula, such as *Lactobacillus* spp., to initiate fermentation. The mixture is then allowed to ferment for 24–48 h at ambient temperature, driving acidification, starch hydrolysis, and flavor development. The fermentation process significantly enhances the availability of protein and carbohydrates. Mahewu contains essential amino acids such as leucine and arginine, as well as non-essential amino acids such as aspartic acid, glutamic acid, and alanine. Potassium is the most abundant mineral; other minerals detected are sodium, phosphorus, magnesium, calcium, manganese, copper, iron, and zinc [233].

**Table 3:** Several fermented foods from Africa.

Product	Description	Microorganisms Identified	Region of Origin	References
Soumbala	Fermented locust bean néré (*Parkia biglobosa*),	*Bacillus subtilis*, *Bacillus. pumilus*, *Priestia megaterium*, *Bacillus licheniformis*	Burkina Faso	[183]
Bikalga	Fermented roselle seeds (*Hibiscus sabdariffa*)	*Bacillus subtilis*, *Bacillus licheniformis*, *Bacillus cereus*, *Bacillus pumilus*, *Pseudobacillus badius*, *Brevibacillus porteri*, *Lysinibacillus sphaericus*, *Lysinibacillus fusiformis*	Burkina Faso	[195]
Dawadawa	Fermented locust bean	*Bacillus* spp.	Ghana & Nigeria	[234]
Okpehe	Fermented *Prosopis africana* seeds	*Bacillus* spp.	Nigeria	[211,216]
Ugba	Fermented African oil bean	*Bacillus* spp.	Nigeria	[223,224]
Iru	Fermented locust bean	*Bacillus* spp., *Enterococcus* and *Lysinibacillus* spp.	Nigeria, Benin	[228]
Kawal	Fermented *Cassia* leaves	*Bacillus subtilis*, *Propionibacterium* spp.	Sudan	[222]
Sigda	Fermented sesame seed	*Candida* and *Saccharomyces* sp. yeasts and *Pediococcus* sp. (eliminated after the second day of fermentation) and *Streptococcus* sp. (that remain throughout the rest of the fermentation) bacterial species	Kordofan and Darfur provinces of western Sudan	[223]
Furundu	Fermented *karkadè* seeds	*Bacillus* sp.	Sudan	[223]
Umqombothi	Maize–sorghum beer	*Lactobacillus* spp., *Saccharomyces cerevisiae*	Southern Africa	[235]
Burukutu	Alcoholic beverage from sorghum and millet	*Escherichia*, *Staphylococcus*, *Bacillus*, *Lactobacillus*, *Leuconostoc*, *Acetobacter*	Nigeria, Benin, Ghana	[236]
Dégué	Fermented millet dough	*Escherichia*, *Bacillus*, *Lactobacillus*, *Enterococcus*	Burkina Faso	[237]
Ogi	Fermented cereal pudding (maize, sorghum, millet)	*Candida krusei*, *Lactiplantibacillus plantarum*, *Limosilactobacillus fermentum*, *Saccharomyces cerevisiae*, *Acetobacter* spp., *Corynebacterium* spp.	West Africa	[238]
Gari	Fermented cassava–cereal derivative (often combined with maize)	*Leuconostoc mesenteroides*, *Lactobaillus plantarum*, *Bacillus subtilis*, *Candida krusei*	West Africa	[239]
Togwa	Cereal-based lactic beverage (cassava, maize, sorghum, millet)	*Lactobacillus* spp., *Pediococcus pentosaceus*, *Weissella confusa*, *Issatchenkia orientalis*, *Saccharomyces cerevisiae*, *Candida pelliculosa*, *Candida tropicalis*	Eastern Africa	[240]
Bushera	Fermented sorghum beverage	*Lactobacillus*, *Streptococcus*, *Leuconostoc*, *Pediococcus*, *Weissella* spp.	Eastern Africa	[241]
Shamita	Fermented barley beverage	*Lactobacillus* spp.	North-East Africa	[242]
Munkoyo	Fermented maize beverage with root extract	*Weissella* spp., *Lactobacillus* spp.	Southern Africa	[243]
Mahewu	Fermented maize or sorghum beverage	*Lactobacillus plantarum*, *Lactobacillus fermentum*	Southern Africa	[244]

### 6.2. Fermented Foods and Beverages from Asia

Across East, South, and Southeast Asia, fermentation is applied to cereals, legumes, vegetables, dairy, and fish, often through spontaneous microbial activity or the use of defined starter cultures [245] (Table 4). Cereal-based fermentations such as Japanese *koji* products, Korean makgeolli, Chinese huangjiu, and Indian kanji use molds (*Aspergillus*, *Rhizopus*), yeasts (*Saccharomyces*, *Candida*), and LAB to hydrolyze starches and generate organic acids, ethanol, and bioactive metabolites [246]. Legume fermentations, including Indonesian tempeh, Japanese natto, and Korean doenjang, enhance protein digestibility, reduce antinutritional factors, and increase B-vitamin content [247,248]. Fermentations in kimchi and paocai are dominated by LAB that produce lactic acid, bacteriocins, and health-promoting metabolites [249]. Dairy fermentations, including Indian lassi and Central Asian kumis, improve lactose digestibility and contribute probiotic microorganisms [250]. Beyond their sensory diversity, Asian fermented fish products provide valuable nutrients and bioactive compounds generated during fermentation. A key metabolite is γ-aminobutyric acid (GABA), produced primarily by lactic acid bacteria, which may contribute to potential antihypertensive effects through mechanisms such as vasodilation and modulation of sympathetic activity. Fermentation also enhances protein digestibility, liberates peptides with antioxidant or antihypertensive potential, and may improve micronutrient availability [251]. Halophilic bacteria and endogenous enzymes in hai *pla-ra* and Filipino *bagoong* can generate amino acids and flavor compounds [252].

#### 6.2.1. Koji

Koji is a traditional food developed in ancient China and later disseminated to Japan and Korea, where it was produced by *Aspergillus* spp. (*A. oryzae*, *A. sojae*, *A. luchuensis* mut. *kawachii*, *A. tamarii*) are cultivated on steamed cereals and legumes [253]. Unlike the mixed amylolytic starters used across Southeast Asia, koji represents a highly specialized microbial system characterized by non-aflatoxigenic strains recognized as safe. Its functional relevance stems from the enzymes (proteases, lipases, and pectinases) that drive efficient saccharification, proteolysis, and flavor formation [254]. In addition to enzymatic activities, koji contains diverse bioactive metabolites, including phenolic acids, hydroquinones, soyasaponins, flavonoids, lysophospholipids, and dipeptides, which contribute to nutritional enhancement, antioxidant potential, and sensory complexity [236,237,238,239,240,241,242,243,244,245,246,247,248,249,250,251,252,253,254,255].

#### 6.2.2. Miso

Miso is fermented by *Aspergillus oryzae* and associated microbial consortia. From a macronutrient perspective, miso provides protein (≈9 g/100 g), complex carbohydrates (≈37 g/100 g), and a modest amount of lipids (≈2 g/100 g), largely in the form of polyunsaturated fatty acids (≈1.85 g/100 g), including linoleic and α-linolenic acids. Its micronutrient profile includes potassium, calcium, iron, and B vitamins, while its sodium content is naturally high due to salt-dependent fermentation [256]. Fermentation enhances the nutritional value of soybeans by generating bioactive peptides, increasing the bioavailability of isoflavones, and improving the digestibility of proteins and carbohydrates. These biochemical transformations contribute to miso’s recognized antioxidant capacity, anti-inflammatory potential, and metabolic benefits, including improved lipid handling, reduced oxidative stress, and modulation of inflammatory markers such as IL-6, CRP, and IL-18, as documented in clinical and experimental studies [256].

#### 6.2.3. Tempe

Tempe is a traditional Indonesian fermented soybean product developed through the long-standing ethno-microbiological practices of Javanese communities, relying primarily on the domesticated mold *Rhizopus oligosporus*. The production involves soaking, dehulling, cooking, and incubating soybeans for 24–48 h, during which *Rhizopus* mycelia bind the cotyledons into a compact, white cake. Although several *Rhizopus* species (*R. oligosporus*, *R. delemar, R. oryzae*, *R. stolonifer*, *R. arrhizus*, *and R. chinensis)*, *R. oligosporus* confers superior sensory and nutritional qualities [257]. Fermentation process produces degradation of mono, di-, and oligosaccharides and polyol compounds that create flatulence [258], enrichment of vitamin B12, attributed mainly to *Saccharomyces cerevisiae*, phytoestrogen production due to *Klebsiella pneumoniae*, including daidzein, genistein, genistin, daidzein, daidzin, and glycitein [259], and immunomodulatory peptides generated predominantly through the enzymatic activity of *Rhizopus oligosporus* [260]. The combination of these events contributes to improved gastrointestinal tolerance [261], antimicrobial activity [262], modulation of the gut microbiota, and potential neurophysiological and cardiometabolic benefits [257].

#### 6.2.4. Natto

Natto, a traditional fermented soybean product produced by *Bacillus subtilis* var. *natto*, is a nutrient-dense food with significant medicinal value. During fermentation, soybean proteins and polysaccharides undergo extensive enzymatic hydrolysis, generating short-chain peptides, free amino acids, and oligosaccharides that are more readily absorbed [263]. Fermentation markedly increases the levels of proteins, cellulose, minerals (calcium, potassium, iron), and vitamin B2 compared with raw soybeans. Fermentation also solubilizes otherwise insoluble soy proteins and promotes the de novo synthesis of digestive enzymes produced by natto bacteria, thereby enhancing gastrointestinal absorption [264]. A key functional metabolite is nattokinase, a fibrinolytic enzyme responsible for natto’s thrombolytic activity [265]. Nattokinase can degrade fibrin, inhibit platelet aggregation, and stimulate endogenous fibrinolytic pathways [266]. Natto also exhibits a broad spectrum of functional activities, including neurological benefits [267], supported by its capacity to degrade amyloid-β peptides, a mechanism relevant to Alzheimer’s disease [268], as well as antibacterial [269] and antitumor properties [270]. Its high vitamin K2 content contributes to bone metabolism and anti-osteoporotic activity [271], while oligosaccharides and γ-polyglutamic acid promote gut health [272]. Moreover, natto can have immunomodulatory and anti-inflammatory activities [273] and reduce the risk of dysglycemia and cardiovascular disease by decreasing the postprandial glucose response in the early phase [266,274]. Finally, the natto’s antioxidant [275], antihypertensive [276], and promotion of gut health via oligosaccharides and γ-polyglutamic acid [272] were confirmed by in vivo studies using animal models.

#### 6.2.5. Doenjang

Doenjang is a traditional Korean fermented soybean paste produced by fermenting *Meju* with *Bacillus subtilis* and molds such as *Mucor*, *Rhizopus*, and *Aspergillus oryzae*. Soybean blocks are formed after soaking, cooking, crushing, and shaping the soybeans, then brining them for several months [277]. The quality, flavor, and nutritional value of Doenjang depend strongly on fermentation conditions, raw materials, and the associated microbial community. Incorporating new ingredients or selecting superior soybean varieties can further enhance its sensory and functional properties [278]. Doenjang is known for its antioxidant, anti-inflammatory, anticancer, and antimutagenic activities [279] as well as its hypocholesterolemic properties [277]. γ-polyglutamic acid produced by *Bacillus* species in doenjang exhibits notable antioxidant and antihypertensive activities in both in vitro and in vivo models. Doenjang is also enriched in isoflavones such as genistein and daidzein, which have demonstrated estrogenic and anticancer effects in cell-based assays and limited human studies. Furthermore, bioactive peptides generated during soybean fermentation show ACE-inhibitory activity, supporting their potential role in blood pressure regulation [280,281].

#### 6.2.6. Kimchi

Kimchi is a traditional Korean fermented vegetable food characterized by a complex matrix of probiotics, prebiotics, postbiotics, and fermentation-derived bioactive compounds [24]. Its fermentation is driven primarily by heterofermentative lactic acid bacteria, including *Leuconostoc*, *Weissella*, and *Lactobacillus*, which contribute to flavor development and functional properties. Kimchi contains diverse phytochemicals, such as isothiocyanates, indole-3-carbinol, allyl sulfides, carotenoids, flavonoids, tocopherols, and polyunsaturated fatty acids, along with vitamins, minerals, and dietary fiber. These components collectively confer antioxidant and immune-enhancing effects [282,283], antiaging [284], anticancer [285,286], and anti-obesity properties [287]. Experimental and clinical studies have demonstrated that kimchi and its LAB strains modulate lipid metabolism and reduce adiposity [288], improve gut microbiota composition by modulating cytokines and inflammatory factors [289], and exert antimutagenic and anticancer activities [290].

#### 6.2.7. Yogurt

Yogurt is a fermented dairy product obtained through the controlled action of *Streptococcus thermophilus* and *Lactobacillus delbrueckii* subsp. *bulgaricus*, which converts lactose into lactic acid and gradually decreases the pH of milk, leading to the formation of a soft, hydrated casein network that entraps whey proteins and water [291,292]. Heat treatment prior to fermentation denatures 90–99% of whey proteins, promoting their interaction with casein micelles and contributing to the characteristic gel structure of yogurt [293,294]. This slow acidification process prevents the abrupt coagulation typical of gastric acid exposure and results in a homogeneous, mesh-like matrix with distinct physical properties compared with raw milk [295]. Nutritionally, yogurt retains the high-quality protein profile of milk, which is rich in essential amino acids and composed of casein and whey in an approximately 8:2 ratio. At the same time, fermentation partially hydrolyzes these proteins, generating bioactive peptides and enhancing digestibility [296,297,298]. Functionally, yogurt provides not only readily absorbable protein but also probiotic and prebiotic components derived from viable and non-viable bacterial cells, metabolites, and fermentation-derived compounds. These include short-chain fatty acids, polysaccharides, and branched-chain hydroxy acids, which contribute to improved gut health, modulation of inflammation, and metabolic benefits such as enhanced glucose regulation [299].

#### 6.2.8. Paocai

Paocai is a traditional Chinese fermented vegetable product prepared from cabbage or radish and seasoned with spices. Paocai possesses high nutritional value, providing abundant vitamins, minerals, dietary fiber, and other functional constituents. These components not only meet essential nutritional requirements but also modulate intestinal microbiota, reduce serum cholesterol, and exert antioxidant, weight-management, and other health-promoting effects [300]. Its spontaneous high-salt fermentation is driven mainly by native LAB, which convert sugars into volatile and non-volatile metabolites that determine flavor and quality. The reliance on natural microbiota leads to variability in sensory properties. To achieve controlled fermentation, selected LAB starters, such as *Lactiplantibacillus brevis*, *Lactiplantibacillus pentosus*, *Leuconostoc mesenteroides*, and *Weissella cibaria*, are increasingly used [301]. Among them, *Levilactobacillus brevis* L., an obligatory heterofermentative species, exhibits probiotic traits, acid tolerance, strong cell-surface hydrophobicity, and substantial auto-aggregation capacity, supporting its survivability and colonization potential. Its cell-free ethyl acetate extract shows broad-spectrum antibacterial and antifungal activity, accompanied by marked inhibition of biofilm formation and reductions in violacein, prodigiosin, and pyocyanin. Metabolomics analyses (FTIR, HPTLC, HR-LCMS) have revealed a diverse array of secondary metabolites, peptides, fatty acids, and phenolic compounds [302].

#### 6.2.9. Lassi

Lassi is a traditional Punjabi fermented dairy beverage, typically made from buffalo milk. It may be prepared in sweet or salted forms and enriched with fruits, spices, or herbs. Although chemical acidification is sometimes used, the best sensory quality is obtained using reconstituted milk fermented with lactic cultures and supplemented with water and sugar [303]. Variants such as bhang lassi and herb- or spice-based (antro, cumin, or black pepper) are common across the Indian subcontinent [304]. Regular consumption of lassi provides multiple nutritional and functional benefits. It supports probiotic intake, digestion, immune function, weight management, bone health, and reduces bloating. Its high digestibility and efficient nutrient absorption make it an excellent vehicle for fortification. Lassi supplies essential and non-essential amino acids, moderate amounts of calcium and phosphorus, and is less acidic than fruit juices [250]. Seven *Lactobacillus* strains (MS001–MS007) were isolated and identified through morphological, biochemical, and PCR-based molecular characterization from Lassi. All isolates showed no hemolysis and were sensitive to most tested antibiotics. They exhibited strong tolerance to gastrointestinal stressors, pH 3.0, bile salts (0.3%), pancreatin (0.5%), and lysozyme, and showed phytase activity, antioxidative capacity, and the ability to form biofilms. Among them, strain MS007 displayed the highest bile salt hydrolase activity, while MS005 showed superior phytate degradation [305]. Probiotic cultures contribute to intestinal health, lactose digestion, and reductions in blood pressure and cholesterol. Its functionality can be further enhanced by adding probiotic strains or herbal extracts. Fiber enrichment helps lower LDL cholesterol, while fruits such as mango can increase vitamin A content and consumer acceptance. Ingredients such as turmeric, ginger, and carrot can boost antioxidant capacity [250].

#### 6.2.10. Pla-ra

Pla-ra is a traditional Thai salt-fermented fish paste. It is produced from freshwater fish mixed with 17–20% salt and a small proportion of roasted rice or rice bran fermented for 6–24 months with *Lactobacillus*, *Pediococcus*, *Weissella*, *Enterococcus*, *Aerococcus*, *Tetragenococcus*, and *Leuconostoc* spp. During this extended maturation, endogenous fish enzymes and halotolerant microorganisms drive extensive proteolysis, generating free amino acids and bioactive peptides that enhance both nutritional value and sensory properties [306]. The addition of roasted rice or rice bran provides fermentable carbohydrates that support mild lactic fermentation, contributing to flavor development and improved digestibility [307]. Pla-ra is a rich source of umami-enhancing amino acids, particularly glutamic and aspartic acids, along with essential amino acids [308] and bioactive compounds that exhibit antihypertensive (via angiotensin-I-converting enzyme inhibition) and antioxidant activities [309].

#### 6.2.11. Bagoong

Bagoong is traditionally prepared in the Philippines through a controlled-salt fermentation process in which small fish (e.g., *Stolephorus* spp., *Sardinella* spp., and *Decapterus* spp. herring, anchovy) or *Atya* spp. or their roe are mixed with 25% salt and allowed to undergo long-term enzymatic and microbial degradation [154]. The early stages of bagoong fermentation are characterized by the presence of Gram-negative rods originating from the raw fish and handling environment. As salting progresses and osmotic pressure increases, these microorganisms rapidly decline due to the high sensitivity of marine bacteria to hypertonic conditions. Protein hydrolysis during fermentation is driven by both endogenous intestinal enzymes and microbial proteases, particularly those produced by *Bacillus subtilis* and *Bacillus coagulans*. These enzymes catalyze deamination and decarboxylation reactions, generating low fatty acids, amides, and other volatile compounds that contribute to the characteristic aroma and flavor of the final product [310]. Throughout fermentation, *Bacillus pumilus* is the dominant species. In the early fermentation phase, *Bacillus megaterium*, *Bacillus coagulans,* and *Bacillus subtilis* also contribute to substrate degradation. As fermentation progresses, the microbial community shifts toward *Bacillus licheniformis*, *Micrococcus* spp. (*Micrococcus colpogenes, Micrococcus varians, Micrococcus roseus),* and *Staphylococcus* species, which participate in the later stages of flavor development and stabilization of the fermented matrix [310]. Bagoong shows a moderate nutrient density, with 15.25% protein, 1.90% fat, and relatively low mineral levels (potassium 80.39 mg/L, magnesium 176.01 mg/L, calcium 3.64 mg/L, sodium 3.21 mg/L, phosphorus 280.96 mg/L). Its lipid fraction contains 2.6% α-linolenic acid (n-3), 0.2% docosahexaenoic acid (n-3), and 4.5% linoleic acid (n-6) [311].

**Table 4:** Several fermented foods from Asia.

Product	Description	Microorganisms Identified	Region of Origin	References
Koji	Fermented rice, barley, or soybeans	*Aspergillus oryzae*, *Aspergillus sojae, Aspergillus luchuensis* mut. *kawachii*, *Aspergillus tamarii*	China, Japan, and Korea	[253]
Miso	Mold-fermented soybean paste	*Aspergillus oryzae*, *Clavispora lusitaniae*, *Meyerozyma guilliermondii*; *Bacillus velezensis*, *Bacillus subtilis*, *Enterococcus durans*, *Rothia kristinae*, *Lactiplantibacillus plantarum*, *Leuconostoc* spp., *Pediococcus* spp., *Weissella* spp.	Japan	[256]
Tempe	Fermented soybean	*Rhizopus microsporus* variety *oligosporus* (*Rhizopus oligosporus*), *Rhizopus oryzae, Rhizopus delemar, Rhizopus arrhizus, Rhizopus stolonifer, and Rhizopus chinensis,* along with *Mucor* spp. and other deuteromycete fungi and yeasts	Indonesia	[257]
Natto	Fermented soybean	*Bacillus subtilis*	China	[24,266]
Kimchi	Fermented Napa cabbage, radish, red chilies, garlic, fish, and salt	*Lactobacillus, Leuconostoc*, and *Weissella*	Corea	[287]
Poacai	Fermented cabbage or radish	*Levilactobacillus brevis*, *Lactiplantibacillus plantarum*, *Lactiplantibacillus pentosus*, *Leuconostoc mesenteroides*, and *Weissella cibaria*		[301]
Yogurt	Fermented milk	*Streptococcus thermophilus* and *Lactobacillus delbrueckii* subsp. *bulgaricus*	Mesopotamia, Anatolia, Iran, and Central Asia	[312]
Lassi	buffalo milk	*Limosilactobacillus fermentum* MS005, *Lactiplantibacillus plantarum* MS007	Punjabi	[305]
Pla-ra	Channa striata(Striped snake head fish, Chon), Trichogastertrichopterus (Gourami, Kra-dee), T. leeri (Kra-dee-nang), Cyclocheilichthys repasson (SilverCarp, Soi), Puntius gonionotus (Barb, Ta-pien).	*Lactobacillus*, *Weissella*, *Pediococcus*, *Enterococcus*, *Tetragenococcus*, *Aerococcus,* and *Leuconostoc* spp.	Thailand	[313]
Bagoon	*Stolephorus* spp., *Sardinella* spp., and *Decapterus* spp. herring, anchovy) or *Atya* spp. or their roe are mixed with 25% salt and allowed to undergo long-term enzymatic and microbial degradation	*Bacillus megaterium*, *Bacillus coagulans, Bacillus subtilis, Bacillus licheniformis*, *Micrococcus colpogenes, Micrococcus varians, Micrococcus roseus,* and *Staphylococcus*	Philippines	[165]

### 6.3. Fermented Foods and Beverages from Latin America

Latin American fermented foods and beverages rely on region-specific raw materials, including maize, cassava, tubers, fruits, vegetables, and animal products (Table 5). Although many countries prepare comparable products, differences in names, processing steps, and seasonings reflect the continent’s cultural and ecological diversity [314]. Because knowledge was transmitted orally, and because ingredient availability varies by geography and season, multiple recipe variants have emerged over time [315]. Solid fermented foods made from native ingredients remain central to Indigenous culinary identity, whereas other fermented items, such as cheeses and cured sausages, were introduced later through European immigration and are not considered traditional [316]. Fermented beverages, both alcoholic and non-alcoholic, also hold cultural importance. Alcoholic drinks are used in ritual or spiritual functions in Indigenous groups, while recreational consumption is more common in industrialized settings. Many traditional beverages are obtained from LAB, which often coexist symbiotically with yeasts. Yeast metabolites stimulate LAB growth, and bacterial acidification enhances yeast activity [317,318]. A wide range of beverages is produced from cassava (*Manihot esculenta*) and maize (*Zea mays*), with fermentation time and alcohol content varying by region. *Chicha* exemplifies this diversity, as it encompasses numerous local variants differing in raw materials, alcohol levels, and preparation methods [319]. In some Brazilian tribes, beverages such as caxiri ferment for up to five days to increase ethanol content. Dominant bacterial genera in the production of caium, calugi, and chicha include *Lacticaseibacillus, Lactobacillus, Lactiplantibacillus*, *Limosilactobacillus*, *Streptococcus*, *Leuconostoc*, and *Weissella* [315]. In several Indigenous groups, chewed sweet potato is used as an inoculum; its endogenous amylases and associated microbiota convert starch into fermentable sugars, enabling the production of beverages such as calugi and caium [314].

#### 6.3.1. Chicha

Chicha is a traditional fermented beverage produced by Indigenous communities across South America. It typically contains 2–12% (*v*/*v*) alcohol and is most commonly prepared from maize, although rice, peanut, cassava, banana, cottonseed, and carob may also be used. It is consumed during religious, agricultural, and social events. In Peru, chicha de jora is produced from local maize varieties and has a low alcoholic content (1–3%). Traditional fermentation historically relied on saliva as a source of amylases, though this practice has increasingly been replaced by alternative enzymatic approaches [320]. Studies on commercial chichas from Peru revealed diverse microbial communities dominated by *Lactobacillaceae*, with *Leuconostocaceae*, *Acetobacteraceae*, *and Streptococcaceae* prevailing in specific formulations [321]. In non-commercial maize chichas from Argentina, bacterial communities differ between production sites: *Lactiplantibacillus plantarum*, *Weissella viridescens, Leuconostoc lactis*, and *Furfurilactobacillus rossiae* predominate in Maimará, whereas *Enterococcus faecium*, *Enterococcus hirae, Leuconostoc mesenteroides*, and *Weissella confusa* dominate in Tumbaya. Yeast communities are predominantly composed of *Saccharomyces cerevisiae*, with *Fusarium* spp. [315]. In the chica produced by Guarani-Kaiowá in Brazil, *W. confusa,* and *Candida bohaiensis* were identified as the predominant bacterial and yeast species, respectively [322]. Other regional variants include rice chicha from the Umutina people, dominated by *Lacticaseibacillus casei*, and bacaba chicha, produced from *Oenocarpus bacaba*, where *Enterococcus durans* and *Pichia caribbica* prevail [323]. Cassava-based chicha (*masato*) is common among Amazonian groups and involves boiling, mashing, and inoculating cassava with chewed root before fermenting for 1–3 days [324]. LAB typically dominates fermented cassava chicha, while high-throughput sequencing studies show a shift from *Bacillus* in unfermented material to *Lactobacillus* and *Acetobacter* in fermented samples, with community differences observed between villages, suggesting long-term microbial domestication linked to communal consumption practices [319].

#### 6.3.2. Calugi

Calugi is a traditional non-alcoholic fermented porridge produced by the Javaé Indigenous people of Tocantins, Brazil. It is typically made from corn, cassava, and sweet potato, with rice occasionally used as an additional substrate [314]. Cassava is peeled, washed, grated, and pressed, while corn is soaked and macerated with wooden tools to obtain a coarse flour. The corn paste is mixed with cassava, diluted with water, and cooked for about two hours [325]. Once cooled, the mixture is inoculated with chewed sweet potato, a practice in which saliva provides the fermentative microbiota [326], often dominated by *Lactobacillus* spp. The mixture ferments in open containers at ambient temperature for 24–48 h, producing a creamy, mildly acidic beverage with very low alcohol content. The microbiota of calugi includes lactic acid bacteria, acetic acid bacteria, mesophilic aerobic bacteria, enterobacteria, and yeasts, with *Bacillus* spp. frequently predominant. Fermentation produces a wide range of organic acids, including lactic, fumaric, acetic, citric, malic, succinic, propionic, tartaric, and oxalic acids, along with ethanol and carbon dioxide, which collectively lower the pH to around 4.0 and shape the flavor profile. Additional compounds, including terpenes, butyric acid, and diacetyl, contribute to its characteristic sensory attributes [327].

#### 6.3.3. Cauim

Cauim is a traditional non-alcoholic fermented beverage produced by the Tapirapé Indigenous people of Mato Grosso, Brazil, using substrates such as cassava, rice, maize, peanut, banana, cottonseed, and pumpkin. The raw materials are cooked for about 2 h, cooled, and then inoculated with juice obtained from sweet potatoes chewed by women of the tribe, which provides saliva-borne microorganisms and amylases that initiate starch hydrolysis. Fermentation proceeds for 24–48 h [328]. Studies on non-commercial cauim have shown a diverse yeast community, with *Candida tropicalis* and *Candida intermedia* as predominant species, alongside *Saccharomyces cerevisiae* and *Kluyveromyces lactis*. These yeasts contribute to starch degradation, flavor formation, and increases in vitamin B and free amino acids. Bacterial diversity is dominated by *Lactiplantibacillus plantarum* and *Limosilactobacillus fermentum* in the later stages of fermentation [315].

#### 6.3.4. Pozol

Pozol, also known as *pochotl* or *pozolli* in Nahuatl, is a traditional fermented beverage originating in Tabasco, Mexico, with roots in pre-Hispanic Mayan culture. It is valued for its nutritional properties and consumed widely across different population groups [329]. The process entails nixtamalizing whole corn kernels with calcium hydroxide, grinding the resulting nixtamal into a dough, forming the dough into spherical units, enclosing them in banana leaves, and allowing them to ferment at ambient temperature for 1–4 days. The fermented dough is then suspended in water to produce the final beverage [330]. This drink contains LAB, including *Lactococcus, Enterococcus*, *Leuconostoc, Weissella*, and *Streptococcus*, particularly *Streptococcus infantarius* and *Streptococcus infantarius* 25,124 strains [331]. The nutritional composition of pozol varies according to corn genotype and processing conditions [332].

**Table 5:** Several fermented foods and beverages from Latin America.

Product	Description	Microorganisms Identified	Region of Origin	References
Chicha	Fermented rice, peanut, cassava, banana, cottonseed, and carob beverage	*Enterococcus*, *Lactococcus*, *Streptococcus*, *Weissella*, *Leuconostoc*, *Lactobacillus*, *Saccharomyces.*	Argentina	[331,333]
Calugi	Fermented corn, cassava, and sweet potato	*Lactobacillus mesenteroides, Lactobacillus lactis; Lactobacillus plantarum, Lactobacillus brevis)*. *Bacillus* sp., *enterobacteria,* and *mesophilic aerobic bacteria* *Fructilactobacillus Rossiae, Weissella viridescens, Weissella confusa, Enterococcus casseliflavus, Enterococcus faecium, Enterococcus mundtti, Enterococcus durans, Enterococcus. hirae, Pediococcus acidilactici,* and *Streptococcus.*	Brazil	[326]
Caium	Fermented cassava, rice, maize, peanut, banana, cottonseed, and pumpkin	*Lactiplantibacillus plantarum* and *Limosilactobacillus fermentum*	Brazil	[315]
Pozol	Fermented maize–cocoa drink	LAB, *Saccharomyces*, *Candida*, *Aspergillus*, *Penicillium*, *Rhizopus*	Mexico	[334,335]

### 6.4. Fermented Foods and Beverages from Europe

European fermented foods and beverages include dairy products such as yogurt, kefir, and a wide range of cheeses, which rely primarily on lactic acid bacteria (*Lactobacillus*, *Lactococcus*, *Streptococcus*) (Table 6). In kefir, there are mixed consortia of yeasts and acetic acid bacteria [336]. Cereal-based fermentations, including sourdough breads and kvass, are driven by stable LAB–yeast associations that contribute to characteristic acidity, texture, and flavor [331]. Vegetable fermentations, such as sauerkraut and pickled vegetables, are dominated by spontaneous LAB communities (*Leuconostoc*, *Lactiplantibacillus*, *Lactobacillus*) [337], whereas fermented meats and fish (e.g., rakfisk) involve LAB, coagulase-negative staphylococci, and salt-tolerant microorganisms [338].

#### 6.4.1. Kefir

Kefir is an ancient fermented beverage originating from the Caucasus region, traditionally produced and consumed for centuries on the northeastern slopes of the Georgian and Caucasus mountains, where kefir grains were historically regarded as a form of family wealth and used to prepare a slightly acidic, low-alcohol drink with recognized health benefits [339,340,341]. Kefir is obtained by inoculating milk with kefir grains, a complex symbiotic consortium of LAB such as *Lactobacillus*, *Lactococcus*, *Leuconostoc*, *and Acetobacter*, as well as yeasts such as *Saccharomyces*, *Kluyveromyces*, *Pichia*, and Candida, embedded in a polysaccharide matrix known as kefiran [342,343,344,345,346]. Traditional milk kefir is produced by adding 2.5–10% grains to pasteurized and cooled milk, followed by 18–24 h fermentation at 20–25 °C, after which grains are removed and reused [347], while water kefir is prepared using sucrose solutions or fruit juices and water kefir grains [348,349]. Fermentation generates a wide spectrum of metabolites, including lactic and acetic acids, ethanol, CO_2_, bioactive peptides, exopolysaccharides, vitamins, and organic acids, which shape kefir’s sensory, nutritional, and functional properties Nutritionally, kefir provides high-quality proteins (≈2.7–3.1%), partially hydrolyzed caseins and whey proteins, essential amino acids, B-vitamins (B1, B2, B5, B6, B12), vitamins A, K, and C, and minerals such as potassium, calcium, phosphorus, magnesium, iron, zinc, and manganese [350]. Nutritional and lactose content are reduced during fermentation, and β-galactosidase activity increases, making kefir suitable for lactose-intolerant individuals [351]. Kefir exhibits multiple nutraceutical properties, including probiotic activity, with LAB and yeasts surviving gastrointestinal transit and inhibiting pathogens through bacteriocins, organic acids, and antimicrobial peptides [352,353], immunomodulatory effects [354], with reductions in inflammatory markers, antimicrobial, hypocholesterolemic, anti-diabetic, and antioxidant activities [355], and antihemorrhagic potential [356]. Kefir also supports gut health by enhancing the production of short-chain fatty acids, an effect demonstrated in vivo in animal models. [357,358] and improving intestinal barrier function [359]. Furthermore, fortification with plant extracts, juices, or functional ingredients (e.g., flaxseed, tea extracts, pomegranate juice) can enhance antioxidant capacity, microbial viability, and sensory attributes [360]. An in vivo study showed that microorganisms from homemade kefir are safe [18].

#### 6.4.2. Sourdough

Sourdough is traditionally produced by mixing ground cereals, legumes, or pseudocereals with water, allowing spontaneous fermentation to occur and gradually establishing a stable microbial ecosystem composed of LAB and yeasts [361]. The dominant microorganisms include *Lactiplantibacillus plantarum*, *Levilactobacillus brevis*, *Limosilactobacillus reuteri, Companilactobacillus* spp., and yeasts such as *Saccharomyces cerevisiae*, *Kluyveromyces marxianus*, *K. lactis*, *and K. aestuarii*, which contribute to acidification, proteolysis, phytase activity, and dough maturation [361]. Their metabolic activity enhances the nutritional and functional attributes of sourdough bread by reducing phytic acid and thereby improving mineral bioavailability; modulating starch digestibility and lowering the glycemic index; increasing protein digestibility through extensive proteolysis; and improving both the technological and physiological properties of dietary fiber [362].

#### 6.4.3. Kwass

Kvass is traditionally produced through a two-stage fermentation process in which rye-based raw materials (such as rye flour, dried rye bread, or rye malt) are mixed with water, sugar, and yeast, undergoing lactic acid fermentation first, followed by alcoholic fermentation. The final beverage typically contains ≤1.5% alcohol, although prolonged fermentation may raise this to 2.5% or more [363,364,365,366]. The microbial community responsible for kvass production includes *Lacticaseibacillus paracasei, Acetobacter pasteurianus,* and *Saccharomyces cerevisiae*, which together produce organic acids, mild carbonation, and characteristic aromatic compounds [367]. Lactic acid acts as a natural preservative, eliminating the need for pasteurization and contributing to kvass’s stability. Nutritionally, kvass contains organic acids, amino acids, carbohydrates, B vitamins, and folic acid, supporting digestive function and metabolic regulation. Recent developments include enriching kvass with polyphenol-rich botanicals, such as black chokeberry (*Aronia melanocarpa*), whose anthocyanins, proanthocyanidins, and hydroxycinnamic acids enhance antioxidant capacity and functional value [368].

#### 6.4.4. Rakfisk

Rakfisk is a traditional Norwegian fermented fish. The fish is dry-salted and stacked belly-up under pressure in sealed containers. Some producers add small amounts of sugar or a LAB-based starter culture. During maturation, the fish remains fully submerged in brine with a final salt concentration of 4–7% (*w*/*w*). Storage at 3–8 °C for 3–12 months enables a slow fermentation/ripening process that shapes the product’s sensory and microbial profile [369].

**Table 6:** Several fermented foods and beverages from Europe.

Product	Description	Microorganisms Identified	Region of Origin	References
Kefir	Fermentation of milk or plant-based substrates	*Lactobacillus*, *Lactococcus, Leuconostoc*, and *Acetobacter*, *Saccharomyces,* and *Kluyveromyces.*	Caucas	[355]
Sourdough Breads	Fermentation of cereals, legumes, or pseudocereals	*Lactiplantibacillus plantarum*, *Levilactobacillus brevis*, *Limosilactobacillus reuteri*, *Companilactobacillus* spp., and yeasts such as *Saccharomyces cerevisiae*, *Kluyveromyces marxianus*, *Kluyveromyces lactis*, and *Kluyveromyces aestuarii.*	Mediterranean, the Middle East, and Europe,	[362,370]
Kvass	Fermentation of rye flour, dried rye bread, or rye malt	*Lacticaseibacillus paracasei*, *Acetobacter pasteurianus*, and *Saccharomyces cerevisiae*	Eastern and Central Europe	[368]
Rakfisk	salmon	*Latilactobacillus sakei*	Norwegian	[369]

## 7. Discussion

The findings summarized in this review underscore the broad functional potential of fermented foods and probiotics; however, the current evidence base remains fragmented and characterized by substantial methodological heterogeneity. Differences in microbial strains, fermentation parameters, analytical techniques, and outcome measures complicate direct comparisons across studies and limit the ability to draw robust, generalizable conclusions. A major challenge arises from the intrinsic strain-specificity of microbial functions. Even within the same species, genomic variability leads to divergent metabolic outputs, including differences in organic acid production, enzymatic activities, detoxification capacity, and postbiotic profiles. This variability is further amplified by the influence of the food matrix. Identical strains may exhibit distinct growth kinetics, metabolite production, and functional properties when inoculated into dairy, cereal, legume, or vegetable substrates. Matrix-dependent buffering capacity, nutrient composition, and endogenous enzymatic activities contribute to these discrepancies, making it difficult to extrapolate findings from one fermented food system to another. Another limitation concerns the predominance of in vitro and small-scale experimental studies. While these models provide valuable mechanistic insights, they often fail to replicate the complexity of human gastrointestinal physiology, host–microbiota interactions, and interindividual variability. Reported outcomes such as antioxidant capacity, immunomodulation, or toxin degradation frequently rely on non-standardized assays, further hindering cross-study comparability. Human clinical trials remain limited in number, heterogeneous in design, and often underpowered, restricting the ability to establish causal relationships or define effective doses and exposure durations. Advances in high-throughput sequencing have improved the characterization of microbial communities in fermented foods, yet inconsistencies persist in sequencing platforms, reference databases, and bioinformatic pipelines. Many studies do not provide strain-level genomic data, limiting the capacity to link specific genetic determinants to functional traits or safety profiles. This gap is particularly relevant for assessing the presence of antibiotic resistance genes, virulence factors, or undesirable metabolic pathways, which remain insufficiently monitored in traditional and artisanal fermentations. Safety considerations also warrant further attention. Although most microorganisms associated with fermented foods have a long history of safe use, the potential transfer of antibiotic resistance genes, the formation of biogenic amines, and the presence of opportunistic pathogens in spontaneous fermentations represent ongoing concerns. Regulatory frameworks differ substantially across regions, and the absence of harmonized guidelines for microbial strain selection, fermentation control, and product labeling complicates risk assessment and consumer protection. The growing interest in postbiotics and fermentation-derived metabolites highlights the need to distinguish the effects of live microorganisms from those of their metabolic products. This distinction is essential for understanding mechanisms of action, ensuring reproducibility, and developing next-generation functional foods with predictable health outcomes. However, the lack of standardized definitions, quantification methods, and biological assays currently limits progress in this area. These considerations emphasize the need for integrated, multidisciplinary approaches combining microbial ecology, food science, metabolomics, genomics, and clinical research. Harmonized methodologies, strain-resolved characterization, and rigorously designed human studies will be essential to strengthen the evidence base, improve reproducibility, and support the development of safe, effective, and scientifically substantiated fermented foods and probiotics.

## 8. Review Limitation

This review is subject to several limitations. The available literature is characterized by substantial heterogeneity in microbial strains, fermentation conditions, and analytical methods, limiting comparability across studies. Much of the evidence derives from in vitro and animal models, which provide mechanistic insights but limited translational relevance. Human studies remain few, heterogeneous, and often underpowered. Strain-level characterization is frequently incomplete, complicating attribution of specific effects. Finally, although supported by a structured search strategy, the narrative nature of this review does not eliminate the possibility of selection bias.

## 9. Conclusions

The available evidence indicates that fermented foods and probiotics can enhance nutrient bioaccessibility, generate bioactive metabolites, and contribute to the detoxification of chemical and microbial contaminants. However, the strength of these findings is limited by substantial heterogeneity in microbial strains, fermentation conditions, analytical methods, and study designs. Most functional claims are supported primarily by in vitro or small-scale experimental models, while well-controlled human studies remain scarce and often underpowered. Safety considerations, including the presence of antibiotic resistance determinants, biogenic amines, and variability in spontaneous fermentations, require more systematic evaluation. To advance the field, standardized methodologies, strain-level characterization, and integrated multi-omics approaches are needed, together with rigorously designed clinical trials. These efforts will be essential to develop fermented foods and probiotics with reproducible, evidence-based functional properties and clearly defined safety profiles.

## Data Availability

No new data were created or analyzed in this study.
